# Comparative and systems analyses of *Leishmania* spp. non-coding RNAs through developmental stages

**DOI:** 10.1371/journal.pntd.0013108

**Published:** 2025-05-28

**Authors:** J. Eduardo Martinez-Hernandez, Victor Aliaga-Tobar, Carolina González-Rosales, Rubens Monte-Neto, Alberto J. M. Martin, Vinicius Maracaja-Coutinho

**Affiliations:** 1 Núcleo de Investigación en Data Science, Facultad de Ingeniería y Negocios, Universidad de Las Américas, Santiago, Chile; 2 Centro de Genómica y Bioinformática, Facultad de Ciencias, Ingeniería y Tecnología, Universidad Mayor, Santiago, Chile; 3 Center for Ecology and Evolution in Microbial Model Systems (EEMiS), Linnaeus University, Kalmar, Sweden; 4 Biotecnologia Aplicada ao Estudo de Patógenos, Instituto René Rachou, Fundação Oswaldo Cruz, Belo Horizonte, Minas Gerais, Brazil; 5 Laboratorio de Redes Biológicas, Centro Científico y Tecnológico de Excelencia Ciencia & Vida, Fundación Ciencia & Vida, Santiago, Chile; 6 Escuela de Ingeniería, Facultad de Ingeniería, Universidad San Sebastián, Santiago, Chile; 7 Advanced Center for Chronic Diseases (ACCDiS), Facultad de Ciencias Químicas y Farmacéuticas, Universidad de Chile, Santiago, Chile; 8 Centro de Modelamiento Molecular, Biofísica y Bioinformática (CM^2^B^2^), Facultad de Ciencias Químicas y Farmacéuticas, Universidad de Chile, Santiago, Chile; 9 Unidad de Genómica Avanzada (UGA), Facultad de Ciencias Químicas y Farmacéuticas, Universidad de Chile, Santiago, Chile; Centro de Pesquisa Gonçalo Moniz-FIOCRUZ/BA, BRAZIL

## Abstract

*Leishmania* spp. is the etiological agent of leishmaniases, neglected diseases that seek to be eradicated in the coming years. The life cycle of these parasites involves different host and stress environments. In recent years, many studies have shown that several protein-coding genes are directly involved with the development and host interactions. However, little is still known about the role of non-coding RNAs (ncRNAs) in life cycle progression. In this study, we aimed to identify the genomic structure and function of ncRNAs from *Leishmania* spp. and to get insights into the repertoire of ncRNAs (RNAome) of this protozoan genus. We studied 26 strains corresponding to 16 different species of *Leishmania*. Our RNAome analysis revealed the presence of several ncRNAs that are shared among different species, allowing us to differentiate between subgenera as well as between species that are canonically related to visceral leishmaniasis. We also studied co-expression relationships between coding genes and ncRNAs which in the amastigote developmental stage for *Leishmania braziliensis* and *Leishmania donovani* revealed the presence of miRNA-like transcripts co-expressed with several coding genes involved in starvation, survival and histone modification. This work represents the first effort to characterize the *Leishmania* ssp. RNAome, supporting further approaches to better understand the role of ncRNAs in gene regulation, infective process, and host-parasite interaction.

## Author summary

*Leishmaniasis* is a neglected tropical disease caused by *Leishmania* parasites, which undergo complex developmental stages transitioning between insect vectors and mammalian hosts. While considerable research has focused on understanding protein-coding genes and their role in parasite survival and host adaptation, the role of non-coding RNAs (ncRNAs) in regulating these processes remains poorly explored. In this study, we performed a comprehensive genome-wide characterization of ncRNAs across 16 *Leishmania* species, integrating comparative genomics, transcriptomics, and co-expression network analyses. We uncovered a diverse repertoire of ncRNAs, including conserved and species-specific molecules, and identified key ncRNAs co-expressed with genes involved in critical processes such as stress response, membrane dynamics, and cytoskeleton organization. Notably, we revealed ncRNAs potentially regulating the expression of genes like aquaglyceroporins, surface proteins, and heat shock proteins, highlighting their role in parasite survival and stage-specific differentiation. This study underscores the importance of ncRNAs in parasite development, survival, and host adaptation. Our findings open new perspectives for exploring ncRNAs as potential targets for therapeutic interventions against leishmaniasis.

## 1. Introduction

Leishmaniases are a group of diseases caused by parasites of the genus *Leishmania* [1,2]. These pathologies present a wide range of clinical manifestations, from self-healing skin lesions in cutaneous leishmaniasis (CL) to the more severe visceral leishmaniasis (VL), which affects organs such as the liver, spleen, and bone marrow [3,4]. Each year, approximately 1 million new cases are reported globally, with an estimated 20,000 deaths [5].

The *Leishmania* life cycle alternates between invertebrate and mammalian hosts, requiring several morphological changes and biochemical adaptations [6]. Infection begins when metacyclic promastigotes (META) are introduced into the dermis via the bite of a sandfly, a blood-feeding *Diptera* [7]. These metacyclic promastigotes are rapidly internalized by skin resident or recruited phagocytes cells (neutrophils, macrophages, and dendritic cells) [7,8]. Inside host cells, the parasites trigger the maturation and morphological transformation into amastigote (AMA) forms. At this point, the parasites multiply by binary fission. This division leads to the lysis of the cell and induces the dissemination of amastigotes, allowing them to infect other cells [7]. Finally, the cycle is completed when the vector ingests the parasites during a blood meal on an infected host, taking with them macrophages carrying amastigotes, triggering morphological changes to procyclic promastigotes (PRO) in the sandfly midgut [7]. Such a challenging life cycle requires parasite adaptation efforts to survive under nutritional starvation, sudden changes in temperature, pH, and cellular plasticity varying from a free-living 15–30 μm length flagellated form to a 3–6 μm rounded intracellular form. All these can be achieved by a very well-orchestrated post-transcriptionally controlled gene expression and epigenetic events, similar to those regulatory mechanisms already reported in other trypanosomatids [9,10].

*Leishmania* parasites exhibit unique characteristics in gene expression regulation [11,12], including constitutive gene expression [13], polycistronic transcription [14–16], and extensive post-transcriptional regulation [17]. Additionally, studies in *L. donovani* suggest that gene dosage through aneuploidy modulates gene expression [18,19]. However, these studies have largely focused on the regulation of protein-coding genes, with little attention paid to non-coding RNAs (ncRNAs) and their role in parasite development and pathogenesis. As a result, our understanding of ncRNAs in *Leishmania* remains limited.

Non-coding RNAs are untranslated transcripts that participate in modulating multiple biological processes [20], such as gene regulation at transcriptional [21] and post-transcriptional levels [22,23], developmental processes [24] or even in many diseases, such as cancer [25] or pathogenic infections [26]. They can be broadly classified into two main subclasses [25,27,28]. The small RNAs (sncRNAs) (< 200 nt), a subclass that includes regulatory RNAs such microRNAs (miRNAs), small interfering RNAs (siRNAs), piwi-interacting RNA (piRNAs), while the long non-coding RNAs (lncRNAs) (> 200 nt) includes several transcripts known to take place regulating several important processes in eukaryotes, such as genome imprinting [29], splicing [30] and chromatin organization [31]. Many classes of regulatory ncRNAs have been identified in various eukaryotic organisms since the discovery of miRNAs in *Caenorhabditis elegans* in 1993 [32], but their roles in eukaryotic pathogens remain poorly studied [33].

The first genome of *Leishmania major* was sequenced in 2005 [34], leading to numerous studies exploring the genome structure and function at the protein-coding genes level [34–43]. In parallel, studies focused on ncRNAs increased since its first description in *Leishmania* parasites in 2006 [44,45]. These works have been mainly concentrated on the identification of specific ncRNAs classes, such as siRNA [46]; microRNA-like and their regulatory roles [47]; small RNAs derived from tRNAs and rRNAs as regulators of host-pathogen interaction processes [48]; small nucleolar RNAs (snoRNA) and their function in rRNA processing [49]; UTR-associated ncRNAs (uaRNAs) [50]; or identifying lncRNAs and their putative functions [51]. More recently, studies characterizing ncRNA repertoires in *L. braziliensis* have highlighted the potential roles of these molecules in trypanosomatids developmental life cycle stages regulation [52,53]. Understanding the role of ncRNAs significantly enhances our understanding of the molecular biology of *Leishmania* and presents opportunities for the development of innovative therapeutic strategies to address these severe parasitic infections.

This study combines computational, transcriptomic, comparative genomics, and systems biology approaches to provide the first comprehensive characterization of the ncRNAs repertoire (RNAome) in 25 strains from 16 *Leishmania* species. By integrating conserved and unique ncRNAs, we uncovered the intricate regulatory roles of these molecules across the species. In addition, we explored ncRNA expression patterns and their relationship with coding genes. Through co-expression networks analysis, we associated ncRNAs with coding genes, pinpointing important ncRNA-coding RNA pairs that are co-expressed during developmental stages. Our findings underscore the non-coding RNAs as players in regulating gene expression during life cycle changes, revealing their essential role in the processes of parasite development, survival, and host adaptation. Furthermore, this work enhances our comprehension of the molecular biology of *Leishmania* and opens new ways for the development of strategies, aimed at targeting ncRNA-driven mechanisms in the fight against leishmaniasis.

## 2. Methods

### 2.1 Databases and datasets

To comprehensively analyze the ncRNA repertoire in *Leishmania* spp., we fetched genomic sequences and whole RNA sequencing (RNA-seq) datasets from publicly available repositories. Complete genome sequences for 25 strains representing 16 different *Leishmania* species were downloaded from the NCBI [54] FTP site and TriTrypDB [55] (S1 Table). Associated metadata for each genome, such as the genome size, annotation details, database accession IDs, and completeness metrics like BUSCO scores, are detailed in S1 Table. Publicly available RNA-seq libraries were retrieved from the NCBI Sequence Read Archive platform (SRA) [56] and encompass samples from three *Leishmania* species at different developmental stages: i) *L. braziliensis* M2903 (MHOM/BR/1975/M2903), study accession PRJNA494068 [53], which includes samples from the amastigote, procyclic, and metacyclic stages; ii) *L. donovani* BPK282A1 (MHOM/NP/2002/BPK282), study accession PRJEB15610 [18], with samples representing the amastigote and undifferentiated promastigote stages; and iii) *L. major* Friedlin (MHOM/IL/1981/Friedlin), study accession PRJNA252769 [57], which contains samples for the metacyclic and procyclic stages. S2 Table provides a detailed overview of the RNA-seq data, including the species, strains, developmental stages, biological replicates, number of reads per library, sequencing platform, and associated references.

### 2.2. Predicting the repertoire of ncRNAs in *Leishmania* spp. genomes

To identify the repertoire of ncRNAs in *Leishmania* spp. we applied two different approaches that combine: i) sequence homology and ii) secondary structure searches (use of covariance models). For the sequence homology-approach, we used the set of non-redundant sequences available in the NR2 database (https://nr2.ncrnadatabases.org/). This repository is a web-based portal that indexes 102 public ncRNA databases, providing a centralized and user-friendly platform to retrieve ncRNAs sequences from different organisms. The database is organized by RNA family, data source, content, and search mechanisms [58]. After downloaded all eukaryotic ncRNAs FASTA sequence available in the NR2 database (FASTA file available in our GitHub, https://github.com/networkbiolab/ncRNA_leish), we aligned these sequences against each *Leishmania* genome using Bowtie2 v2.3.5.1 [59]. We selected as option the report of all alignments (*-a* in command) and allowed 1 mismatch (*-N* 1). This step will enable us to align local to local, therefore predicting ncRNAs with a ≥ 80% sequence similarity, and in consequence, also allow us to recover the specific genome region where sequence homology between ncRNA sequences and *Leishmania* genomes were detected. Next, BED files containing coordinates location (loci) of predicted ncRNAs on the *Leishmania* genomes of were obtained using SAM2BED tool from BEDOPS v2.4.41 with default parameters [60]. Additionally, to the NR2 database, we also utilized all previously described non-coding RNAs (ncRNAs) from *Leishmania* species deposited in TriTrypDB. We selected all genes annotated with the attribute “ncRNA_gene.” A manual curation step was subsequently performed to eliminate ncRNAs erroneously tagged as ncRNAs (error in gene ID name) from the dataset. Finally, the curated ncRNA dataset was used to conduct homology searches across all genomes analyzed in this study, applying same steps and parameters previously used for NR2 searches.

The second approach involved the use of covariance models, a statistical model used to describe the conserved sequence and secondary structure of RNA molecules. To do so, we used StructRNAFinder pipeline [61], which integrates Infernal v1.1 [62], RNAfold from ViennaRNA v2.7.0 [63] and Rfam v14.1 [64] to predict and annotate ncRNAs families in each *Leishmania* genome. An e-value cut-off of 0.001 was applied for cmsearch. A score of 10 was used to identify and annotate RNA families with StructRNAfinder, as reported by Torres et al (2017), with *L. braziliensis* [52]. Similar to the previous approach, this allowed us to predict ncRNAs on genome regions, but using secondary structure instead of sequence homology.

The merge of BED files generated by both approaches was performed using MergeBed from BEDtools [65]. Noteworthy, this step allowed us to remove redundancies, i.e., ncRNA prediction in the same locus. Intragenic ncRNAs were filtered out using intersectBED [65]. The final set of ncRNA sequences in FASTA format was obtained using the final BED file that contained the complete ncRNA repertoire of each *Leishmania* genome through BEDtools GetFasta [65]. GTF files for predicted ncRNAs were built using AGAT toolkit [66]. RNA class annotation for predicted ncRNAs were determined by consensus between the classifications identified by both predictions.

### 2.3. Transcriptional evidence of predicted ncRNAs

Transcriptional evidence for predicted ncRNAs was performed through RNA-seq data analysis. The public available data of: i) *Leishmania braziliensis* MHOM/BR/1975/M2903*,* ii) *Leishmania donovani* BPK282A1 (MHOM/NP/2002/BPK282A1) and iii) *Leishmania major* Friedlin (MHOM/IL/1981/Friedlin) were download and used for subsequent analyzes, according to previously described parameters [48]. Briefly, the RNA-seq mapping and read counts measuring, were performed with the following modifications. FastQC v0.11.8 (https://www.bioinformatics.babraham.ac.uk/projects/fastqc/) and Fastp v0.23.4 [67] were used to evaluate and filter out low quality reads from RNA-seq data, considering a Phred cut-off value of Q = 30. The infer_experiment.py tool from RSeQC v5.0.4 [68] was applied to determine the strandness of the reads. High-quality reads were then mapped to genomes using Bowtie2 (with *-N* 1 *--local* parameters) [59]. Read count values for the predicted ncRNAs from the three *Leishmania* species were calculated using featureCounts from Subread v2.0.8 [69] with parameters for paired-end reads (*-p*) and strand-specific reads (*-s*). Then, the genes counts were normalized using FPKM (Fragments Per Kilobase per Million mapped fragments). A modest filter of ≥ 1 FPKM was selected to determine a ncRNAs as expressed, comparable to other works with similar methodology [70–73].

### 2.4. *Comparative analysis* of *ncRNAs*

Conservation analysis of ncRNA repertoire in *Leishmania* spp., was carried out. First*,* predicted ncRNA sequences of all species were clustered using CD-HIT version 4.8.1 [74] with a threshold of 85% of sequence similarity with an alignment coverage of 85% for the shorter sequence and 85% of query coverage. Additionally, we select a global alignment with *-G* parameter and select the best cluster for each sequence using the *-g* parameter. Next, a binary presence-absence matrix was then constructed based on the predicted ncRNA clusters. The set of ncRNA clusters conserved across all species was designated as the “core-RNAome”, while the “accessory RNAome” refers to ncRNA clusters present in some, but not all, *Leishmania* genomes. The total collection of core-RNAome, accessory-RNAome, and species-specific (unique to strains) ncRNA clusters was collectively defined as the “pan-RNAome.

We generate a cladogram to visualize the species clustering based on the ncRNA family distribution by performing hierarchical clustering with Euclidean distance on the presence/absence matrix. We then compared the resulting cladogram with a phylogenetic tree derived from a set of conserved orthologous proteins. For this, we constructed core-genome phylogenetic trees using 613 conserved orthologous proteins from the core-genome of 25 *Leishmania* representatives. We employed a bidirectional best-hit algorithm for orthologs clustering using ProteinOrtho v5.11 [75], with default parameters. Next, conserved orthologous multiprotein family sequences were aligned using MAFFT version v7.453 [76], considering the L-INS-I iterative refinement method. The alignments were masked to remove unreliable aligned regions with GBLOCKS version 0.91b [77] with default parameters. Maximum likelihood trees were prepared for concatenated alignments through IQ-TREE version 1.6.12 [78], using 1,000 replicates as bootstrap with the best-suited substitution model. The final tree was visualized using Figtree (http://tree.bio.ed.ac.uk/software/figtree/).

### 2.5. Differential expression analysis among developmental stages in *Leishmania* spp

The same RNA-seq libraries used in the transcriptional evidence step were employed to perform expression analysis across different developmental stages *L. braziliensis*, *L. donovani* and *L. major*. Differential expression was performed using DESeq2 v1.46.0 [79,80]. First, to account for differences in library sizes and compositional biases across RNA-seq samples, we applied Trimmed Mean of M-values (TMM) normalization using the edgeR package. This method calculates normalization factors by trimming the most extreme count values to reduce the influence of highly expressed genes, which may skew the overall count distribution. This normalization procedure ensures more accurate comparisons of gene expression levels between samples, allowing for reliable downstream analyses such as differential expression testing and co-expression network analysis.

Differentially expressed genes (DEGs) were calculated using DESeq2 and EdgeR with the following thresholds: *p-value* < 0.001, false discovery rate (FDR) or adjusted pvalue < 0.05, and ∣log2FC∣ > 1. We then generated a consensus between both tools to identify candidate genes specifically associated with developmental stages.

### 2.6. Co-expression network analysis

To identify modules related to the developmental stages of *Leishmania*, we performed a gene co-expression analysis using Weighted Gene Co-expression Network Analysis (WGCNA) package v1.69 [81]. Normalized read counts in TMM of coding genes and ncRNAs recovered from RNA-seq data analysis were used as input for WGCNA. A soft threshold power (β) was chosen to achieve a scale-free topology (scale-free R² > 0.85), ensuring that the network structures are biologically meaningful for each *Leishmania* species data. A weighted adjacency matrix was created by raising the absolute value of the Pearson correlation coefficient between gene pairs to the selected soft-threshold power. This adjacency matrix was transformed into a topological overlap matrix (TOM), which measures network connectivity and accounts for shared neighbors between gene pairs. Genes were hierarchically clustered based on TOM dissimilarity, and modules were defined using the dynamic tree-cut algorithm with a minimum module size of 30 genes. Module-stage relationships were determined by calculating Pearson correlation coefficients between modules and developmental stage traits (procyclic, metacyclic, or amastigote stages). Modules with significant correlations (R^2^ > |0.7|, p < 0.05) were considered stage-associated. Next, gene significance (GS) and module membership (MM) metrics were utilized to identify genes that are strongly associated with developmental stages and their corresponding modules. GS measures the correlation between an individual gene expression and the developmental stage of interest. On the other hand, MM quantifies the correlation of a gene expression profile with the eigengene of its module. A gene *X* was considered to be highly associated to developmental stage *Z*, if it met the criteria: X(Z) = GS > 0.70, *p* < 1e-3 and MM > |0.85|, *p* < 1e-5.

To identify hub genes in the coexpression network, we utilized the NetworkAnalyzer plugin v4.5.0 [82] of Cytoscape. The network was analyzed to compute key topological parameters, including degree, betweenness centrality, and closeness centrality. To define hub genes, we applied a threshold based on the 90th percentile of these metrics, selecting nodes with the highest connectivity, influence, and proximity within the network. The final hub gene list was extracted and further examined for potential biological relevance, particularly their associations with developmental stages.

### 2.7. Functional associations of ncRNA in Leishmania parasites

To obtain functional associations for each ncRNA, we employed a modified version of the method described by Liao and collaborators [83]. First, we obtained the Gene Ontology (GO) annotation GAF files from TritrypDB [55], which contain GO term annotations for each coding gene, that include biological process (BP), cellular component (CC), and molecular function (MF). Then, we performed a GO term enrichment first on the first-level coding gene neighbors for each ncRNA obtained from coexpression network analysis using the cytoscape app BiNGO v3.0.5 [84]. The *p*-values were adjusted using the FDR method, with a significance threshold of *p* < 0.05. To reduce redundancy in GO terms, we processed the results using Revigo v1.8.1 [85], which clusters similar terms based on semantic similarity. All code employed in this work are available at GitHub: https://github.com/networkbiolab/ncRNA_leish.

## 3. Results

### 3.1. Dissecting the repertoire of non-coding RNAs across *Leishmania* spp

**Fig 1:**
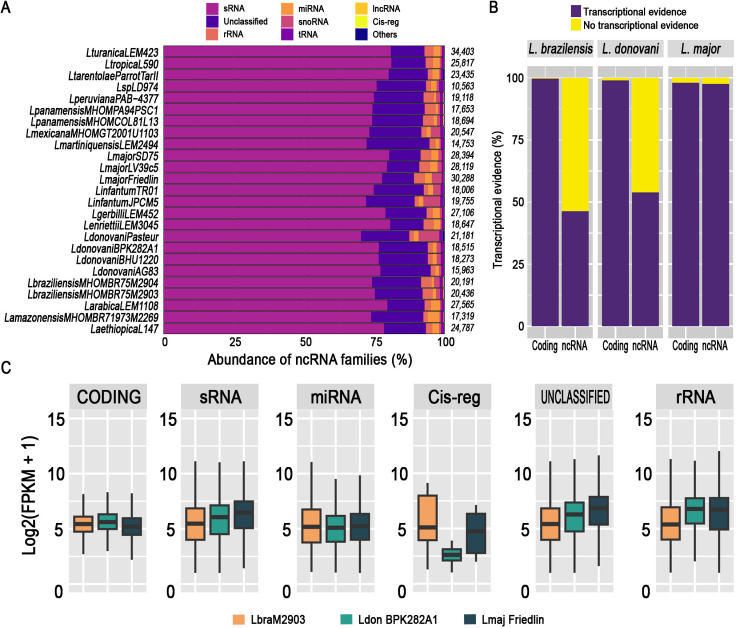
ncRNAs repertoire and transcriptional evidence in *Leishmania* parasites. **A.** Distribution of ncRNAs identified in all species. Those ncRNAs representing less than 0.5% were categorized as “Others”. The presence of a large proportion of non-categorized ncRNAs (unclassified) are elements that could not be assigned to any group due to a lack of biological information related to their secondary structure motifs or functional annotation in the original database. **B.** ncRNAs gene prediction was validated by transcriptional evidence using RNA-seq analysis. We observed expression for a rate between 46.3% (*L. braziliensis*) to 97.55% (*L. major*) of predicted ncRNAs. **C.** Comparative expression values of log_2_ counts per million (log_2 CPM_) normalized read counts. The top five representative ncRNAs according to the number of predictions are represented, together with the coding genes expressed in *L. braziliensis*, *L. donovani*, and *L. major*.

We employed a combined computational approach, utilizing covariance model comparisons of established RNA families, and sequence similarity searches from public databases to characterize the ncRNA repertoire across 25 genomes from 16 different *Leishmania* species. Our analysis, described for the first time, a plethora of different types of ncRNAs (Fig 1A). We predicted a total of 539,528 ncRNAs, with individual species counts ranging from 10,563 (*LspLD974*) to 34,403 (*Leishmania turanica* LEM423). A total of 13 well-known and one unclassified RNA types were identified across the *Leishmania* spp. genomes, based on the NR2 and Rfam [64] original annotations and nomenclature. Additionally, we identified a set of ncRNAs without specific functional annotations but conserved according to other RNA sequences available in public databases and indexed in NR2. The most representative RNA class is sRNA, with an average of 16,622 copies per genome, while the less abundant were scaRNA and SRP, each represented by 38 and 30 ncRNAs, respectively. The top five RNA classes according to their average occurrence across all genomes were: sRNAs (16,622) that represent the 77% of identified ncRNAs on *Leishmania* genomes repertoire (Fig 1A), Unclassified (3,312) representing the 15% from the total, rRNAs (575, the 2.6%), miRNAs (miRNA-like) (453, the 2.1%), and snoRNA (357, the 1.6%). Notably, the ncRNAs presented a GC content ranging between 48% and 60%, and lengths ranging from 18 (miRNA-like) to ~6,000 nt (unclassified RNAs). Further details on the ncRNA repertory for each species are provided in S1 and S2 Tables.

To validate the transcriptional evidence of the predicted ncRNAs, we integrated publicly available RNA-seq data from three different species: *L. braziliensis* M2903 (MHOM/BR/75/M2903) [53], *L. donovani* BPK282A1 (MHOM/NP/02/BPK282) [18], and *L. major* Friedlin (MHOM/IL/81/Friedlin) [57]. We selected a modest expression cut-off of 1 FPKM based on previous works in different samples and model species [70–73], in order to define *bona fide* expressed transcripts. In this sense, we obtained transcriptional evidence for several ncRNAs, ranging from 46.3% in *L. braziliensis* M2904 to 97.55% of the predicted ncRNAs in *L. major Friedlin*. Additionally, we observed expression for approximately 98% of protein-coding genes across these three species using the same RNA-seq datasets (Fig 1B).

Next, we filtered all normalized counts of the most representative ncRNAs classes and compared expression values for ncRNAs and protein-coding genes in each strain (Fig 1C). We noted that in *L. braziliensis*, the mean expression value for ncRNAs (log_2_) was 7.4 FPKM*.* A similar trend was observed for *L. major*, with an average expression of 7.25 FPKM. In *L. donovani*, we found an FPKM average of 6.94.

### 3.2. The *Leishmania* spp. pan-RNAome: ncRNAs conservation across 25 genomes

**Fig 2:**
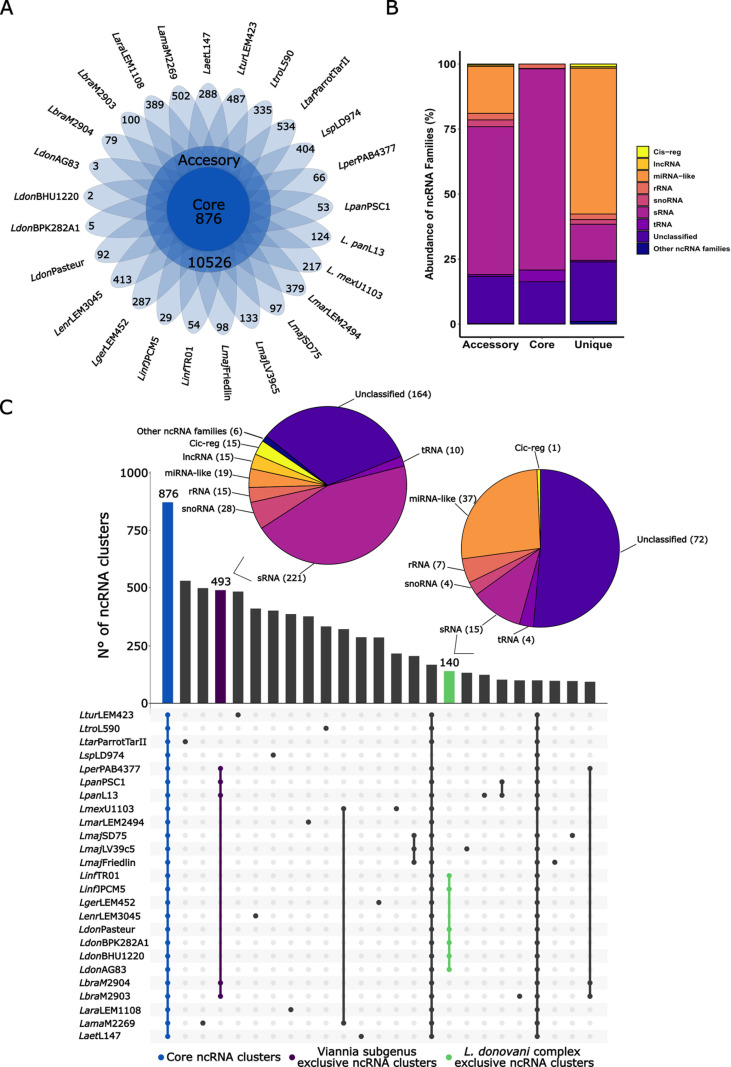
ncRNA conservation analysis through *Leishmania* spp. **A.** Flower map indicating the ncRNA conservation across25 *Leishmania* parasites. The inner circle represents the core-RNAome, while the outer circle displays the accessory-RNAome Each petal represents species-specific ncRNAs identified for each evaluated species. **B.** Abundance of ncRNA families in the pan-RNAome of *Leishmania* spp. **C.** Upset plot corresponding to the top 25 conserved clusters within all 25 genomes. In Purple are represented the clusters conserved exclusively in species of *L. donovani* complex, related to visceral leishmaniasis (VL) (*L. donovani* and *L. infantum* strains), while the clusters associated with *Viannia* subgenus (*L. braziliensis*, *L. panamensis*, and *L. peruviana* species) are in green. The core clusters are in blue. Pie charts for the *Viannia* subgenus and *L. donovani* complex lineages illustrate the relative abundance of different ncRNA classes.

We performed a comparative genomic analysis to explore the ncRNA repertoire across *Leishmania* genomes. After clustering the predicted ncRNAs using CD-HIT [74], we identified 16,572 clusters, which we refer to as the pan-RNAome of *Leishmania* spp. The pan-RNAome was further divided into a core-RNAome, consisting of 876 (5.27%) clusters (Fig 2A), representing a total of 230,788 ncRNAs conserved across all species, and the accessory-RNAome, made up of 10,526 (63.52%) clusters encompassing 30,1971 ncRNAs (Fig 2A). Additionally, we identified 6,552 species-specific ncRNAs (5,170 clusters) in the *Leishmania* genomes, corresponding to 31.21% of the pan-RNAome (Fig 2A).

Conservation within ncRNA types such as, rRNAs, snRNAs, snoRNAs, sRNAs and tRNAs comprised 89.79% of the core-RNAome. The remaining 16.21% consisted of unclassified RNAs (Fig 2B). The species-specific ncRNAs showed greater diversity in ncRNA types compared with core-RNAome, with unclassified RNAs, miRNA-like, and sRNAs being the most abundant classes, representing 92.82% of the total unique ncRNA inventory (Fig 2B). The accessory-RNAome was mainly integrated by unclassified RNAs, miRNA-like, and sRNAs. We also distinguished a subset of 793 ncRNA clusters conserved only in species of the *Viannia* subgenus and absent in other groups (Fig 2C). These *Viannia*-specific clusters were primarily annotated as unclassified ncRNAs, sRNAs and snoRNAs but also, we distinguished that the 10.34% of ncRNAs shared across Viannia subgenus species has potential roles in gene expression regulation such, lncRNAs, miRNA-like, Cis-regulatory RNAs (Fig 2C). Next, we evaluated the ncRNA conservation in species of *L. donovani* complex, canonically associated with visceral leishmaniasis. Based on the literature, we selected *Leishmania donovani* and *Leishmania infantum* as representatives of VL-associated species [1,86,87], identifying a subset of 140 ncRNA clusters (Fig 2C). These clusters were mostly made up of unclassified ncRNAs and miRNA-likes (Fig 2C).

**Fig 3:**
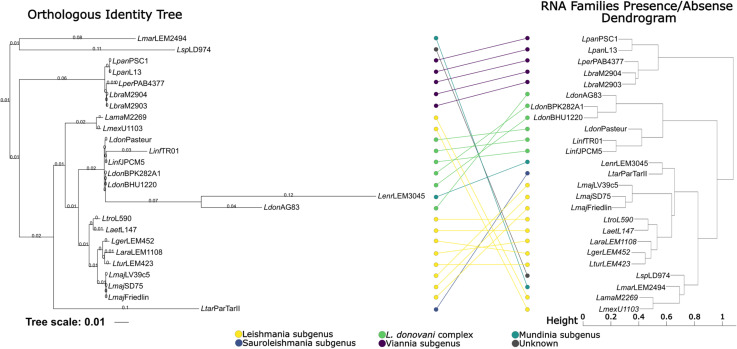
Phylogenetic analysis of the Leishmania genus and comparison with clustering of core RNA families presence/absence. The phylogenetic tree on the left is based on orthologous gene identity comparisons across multiple Leishmania species and subspecies. The dendrogram on the right represents the clustering of RNA families based on the presence/absence of core RNAome elements, calculated using the Jaccard coefficient similarity matrix. Nodes are colored according to Leishmania subgroups: the Leishmania subgenus (yellow), Sauroleishmania subgenus (green), and Viannia subgenus (purple). The tree scale is indicated as 0.01 substitutions per site.

To assess the relationship between phylogeny based on protein-coding genes and the distribution of ncRNA classes across all *Leishmania* species causing different clinical manifestations or from distinct subgenus, we compared the phylogenetic relationships for the primary sequences of 613 core orthologous protein-coding genes conserved within all 25 *Leishmania* isolates, with a clustering generated using the Jaccard similarity index computed from the ncRNA presence/absence matrix. Interestingly, both approaches (sequence phylogeny and Jaccard coefficient clustering) showed similar patterns, indicating a phylogenetic relationship in the conservation and distribution of ncRNAs (Fig 3).

### 3.3. Stage-specific differential expression of coding genes and ncRNAs

**Fig 4:**
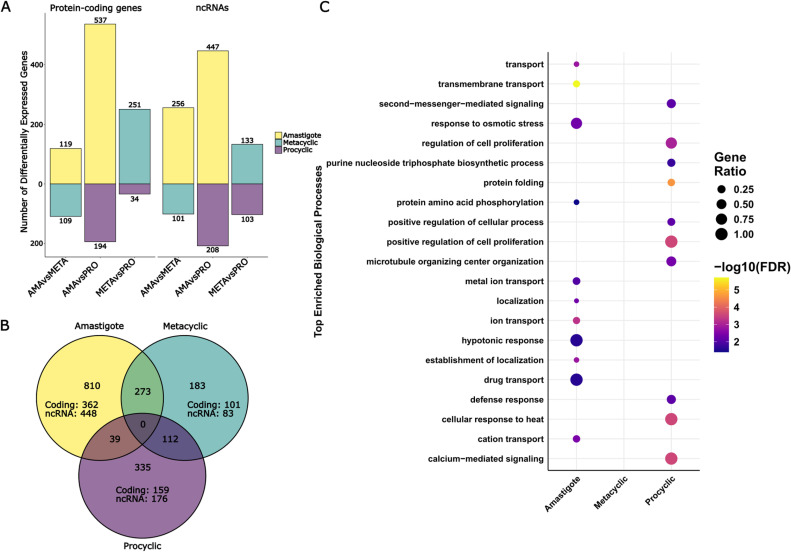
Differentially expressed coding and non-coding genes through developmental stages in *L. braziliensis.* A. Differentially expressed genes in amastigote, metacyclic, and procyclic developmental stages. The number on top of each bar represents all overexpressed genes in each stage comparison. The shadow bar represents the ncRNAs overexpressed. B. Venn diagram highlighting the exclusively DEGs of each developmental stage. C. Top 10 GO biological processes enriched terms (*p*-value) according to the exclusive genes for each developmental stage. Metacyclic stage is left empty to indicate that have not enrichment processes.

We obtained the set of differentially expressed protein-coding genes and ncRNAs across the three main developmental stages of *L. braziliensis*. We analyzed the expression of 5,528 ncRNAs and 8,345 coding genes through pairwise comparisons of the amastigote, metacyclic promastigote, and procyclic promastigote developmental stages. Notably, the amastigote stage exhibited the highest number of DEGs, with 537 protein-coding genes and 447 ncRNAs differentially expressed (DE) when compared to the procyclic stage (Fig 4A), that denoted an extensive transcriptional change during this stage, impacting not only protein-coding genes but also the expression of ncRNAs. In total, 1,103 DEGs were identified in the amastigote, in which 810 were found to be exclusive for this stage (783 mRNAs and 220 ncRNAs). In comparison to the others, 273 genes were shared with the metacyclic, while only 39 genes overlapped with the procyclic, highlighting the profound transcriptional differences between the amastigote and procyclic stages (Fig 4B). In the metacyclic stage, 83 ncRNAs and 101 protein-coding genes were exclusively differentially expressed, whereas the procyclic stage exhibited 176 ncRNAs and 159 coding genes with exclusive differential expression (Fig 4B).

A Gene Ontology (GO) enrichment analysis was performed to identify the biological processes associated with the differentially expressed genes (DEGs) in each developmental stage of *Leishmania braziliensis* (Fig 4C).

In the amastigote stage, we found that DEGs were significantly enriched in biological processes related to transmembrane transport, response to osmotic stress, hypotonic response and establishment of localization.

In contrast, the procyclic stage showed significant enrichment in processes related to protein folding, regulation of cell proliferation, cellular response to heat among others. In this sense, the procyclic stage seems to reflect the other side of the coin of the amastigote, suggesting a completely different transcriptional configuration, according to the limited overlap of differentially expressed genes found between both stages (Fig 4B and S5 and S6 Tables).

### 3.4. Co-expression network analysis identifies developmental stages-associated gene modules in *Leishmania braziliensis*

A total of 13,872 genes (5,528 ncRNAs and 8,345 coding genes) of *L. braziliensis* were employed to construct the weighted gene co-expression networks using WGCNA [81]. The set-up condition by our network construction was a soft threshold power β at 14, the scale-free network fitting index (R^2^) greater than 0.85 was set to ensure low mean connectivity and high scale independence. Ten co-expression modules were recognized in *L. braziliensis*. The number of genes per module varies from 21 (M9) to 2,706 (M0). The total number of genes per module, as well as its composition of coding and ncRNAs is described in detail in S7 Table.

**Fig 5:**
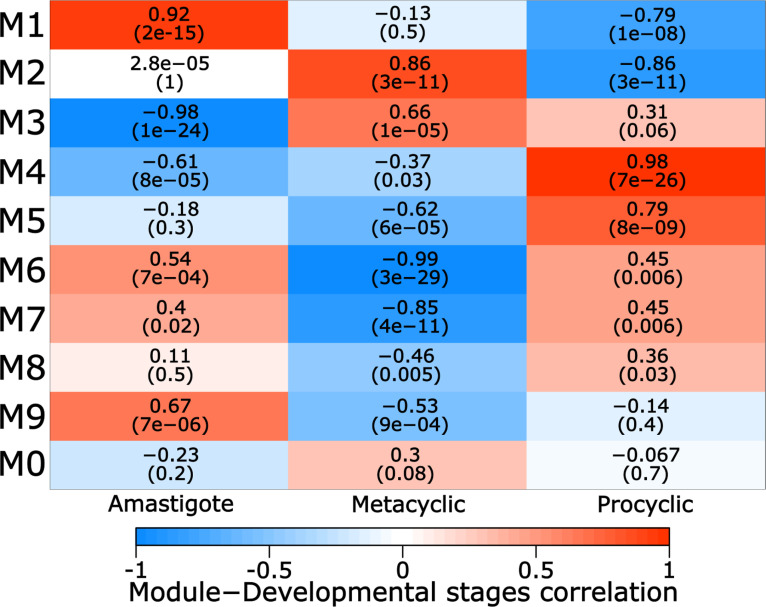
Co-expression module-developmental stages associations for *L. braziliensis.* Each row corresponds to a module and the columns to a developmental stage. Each cell contains the corresponding correlation and *p-value* (in parenthesis). The table is color-coded by correlation according to the color legend. The heatmap shows that modules M4 (R^2^ = 098 and *p* < 0.001), M5 (R^2^ = 0.79 and *p* < 0.001) have the major correlation with the procyclic developmental stage, besides M2 (R^2^ = 0.86 and *p* < 0.001), M3 (R^2^ = 0.66 and *p* < 0.001) are tightly related to the metacyclic stage and modules M1 (R^2^ = 0.92 and *p* < 0.001); and M9 (R^2^ = 0.67 and P < 0.001) are correlated to the amastigote developmental stage.

To analyze the correlation of each module in *Leishmania braziliensis* developmental stages*,* we used a module-development stage relationship comparison. The relationship between co-expression modules and developmental stage is shown in Fig 5. After assessing strong correlations between all modules and developmental stages, we found that module M4 had the highest correlation with the procyclic promastigote (R^2^ = 0.98 and *p* < 0.001). In the same way, module M2 presented a stronger correlation with metacyclic promastigote (R^2^ = 0.86 and *p* < 0.001), and M1 with amastigote (R^2^ = 0.92 and *p* < 0.001).

After identifying the modules related to each developmental stage, we generated a filter based on the Module Membership (MM) and Gene Significance (GS) metrics, obtained from our coexpression analysis, to select the genes most closely associated with each developmental stage. Additionally, we filtered the network to retain only the top 1% of the strongest connections between genes, focusing on the most biologically relevant information for each phase of the *Leishmania braziliensis* life cycle.

Using this approach, we identified 1,042 genes highly correlated with the amastigote stage (68 ncRNAs and 974 coding genes) (S1 Fig). Similarly, we identified 907 genes highly related to procyclic promastigotes, of which 110 were ncRNAs and 797 coding genes (S2 Fig). Additionally, 266 coding genes and 13 ncRNAs were highly associated with metacyclic promastigotes (S3 Fig).

Subsequently, we performed a biological process (BP) GO enrichment analysis on these modules. The module associated with amastigotes showed enrichment in biological processes related to host interaction, such as the “biological process involved in interspecies interaction between organisms.” Other enriched processes included “regulation of autophagy” and “response to osmotic stress” (S8 Table). The enrichment of BP in procyclic promastigotes revealed processes associated with defense, flagellar structure, and cell proliferation (S9 Table). Finally, processes involved in amino acid metabolism and carbohydrate transport were enriched in the module associated with metacyclic promastigotes (S10 Table).

### 3.5. Detection of potential ncRNAs involved in developmental stages of *Leishmania braziliensis*

**Fig 6:**
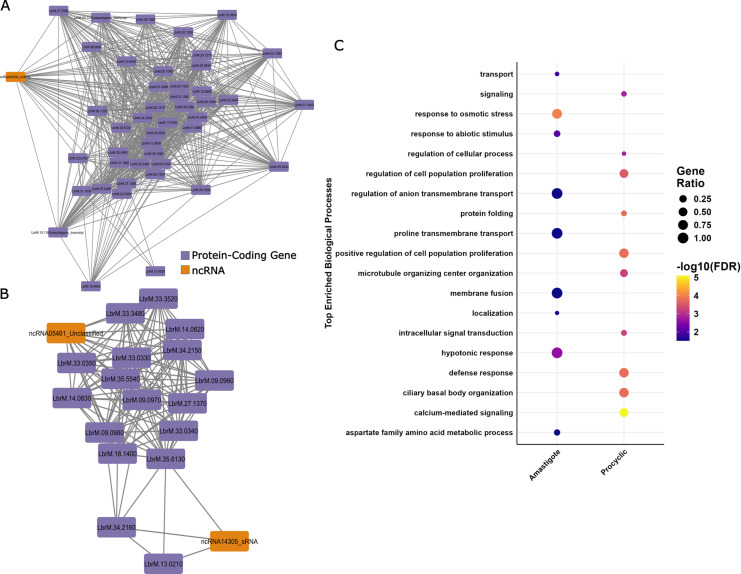
Biological processes GO enrichment of hub ncRNAs related to amastigote and procyclic promastigote developmental stages in *L. braziliensis.* **A** and B. Sub-networks of the hub genes related to amastigotes (**A**) and procyclic promastigores *L. braziliensis* (B). In orange are represented ncRNA genes, while, in purple, the co-expressed protein-coding genes. C. Top 10 biological processes terms assigned to the ncRNAs in each developmental stage based on the co-expressed protein-coding genes.

To assess the possible functions of the ncRNAs associated to developmental stages of *L. braziliens*, differentially expressed mRNAs and ncRNAs were selected to filter the co-expression network corresponding to each developmental stage. Next, we predicted the functions of selected ncRNAs from the co-expression network by combining hub- and module-based methods previously reported [83]. In this analysis, we selected the hub genes based on their key topological parameters, such as degree, betweenness centrality, and closeness centrality, and expression and gene significance metric. Based on this filter, a final set of 45 genes (1 lncRNA and 44 mRNAs) were identified as hub genes in amastigote (Fig 6A). At the same time, the evaluation of the procyclic promastigotes characterized 2 ncRNAs (one unclassified RNA and sRNA) and 17 protein-coding genes as hub (Fig 6B). Otherwise, we identified 8 protein-coding genes as hub in the metacyclic promastigote related module, but no ncRNAs.

Through the guilty by association functional annotation approach, we predicted the possible function of the hub ncRNAs in the amastigote and procyclic promastigote developmental stage. The ncRNA00056_lncRNA a lncRNA gene was co-expressed with 44 protein-coding genes (Fig 5A). Among them, three genes are related to response to osmotic stress. These protein-coding genes are annotated as Aquaglyceroporin 1, encoded by LbrM.31.0020, Vesicle-associated membrane protein 7 (LbrM.27.2560) and the amino acid permease 24 (LbrM.10.0840). Additionally, the expression of ncRNA00056_lncRNA is correlated with the expression of the gene LbrM.27.0480, which encodes the autophagy protein APG9. Our analysis suggests that this lncRNA could participate in biological processes mostly associated with response abiotic stimulus, response to osmotic stress as well as transmembrane transport and amino acid homeostasis (*p*-values < 0.05) (Fig 6C and S11 Table). Interestingly, this lncRNA was conserved with Vianna subgenus species. We also observed that ncRNA14305_sRNA a sRNA was co-expressed with 3 protein-coding genes in procyclic promastigote developmental stage (Fig 6C), These genes encode for the kinetoplastid membrane protein-11 (LbrM.34.2160), kinetoplast-associated protein (LbrM.35.6130) and alpha tubulin (LbrM.13.0210) (Fig 6B). Additionally, we identified that ncRNA05461_Unclassified co-expressed with other 15 protein-coding genes in *L. braziiliensis* procyclic promastigotes (Fig 6B), possessing an enrichment in defense response associated with the gene LbrM.34.2150, that encodes to a kinetoplastid membrane protein-11. Interestingly, this miRNA-like was also co-expressed with several copies of chaperone HSP-83 (LbrM.33.0330, LbrM.33.0340, LbrM.33.0350) and HSP-110 (LbrM.18.1400) (S12 Table).

### 4. Discussion

Here we have described for the first time a genome-wide prediction of non-coding RNAs in 16 *Leishmania* species. Our results on 25 different strains revealed expression patterns of coding and non-coding RNAs highly related to different parasite developmental stages. These findings may suggest a role in the regulation of morphological differentiation in both insect and mammal host stages, as occurs in other Trypanosomatidae [88]. So far, different efforts have been performed to describe the repertoire of distinct ncRNA classes in *Leishmania* parasites [44,46,47,49,50,52,53,89,90]. However, different from these previous works, our computational approach did not focus on any particular ncRNA class, thus allowing an unbiased genome-wide identification of the ncRNA repertoire. We followed a similar approach implemented before by our group to describe the sets of ncRNAs available in *Leishmania braziliensis* [52].

We combined sequence similarity searches and covariance model comparisons to identify and annotate a large number of ncRNAs in all studied genomes. Additionally, we used publicly available RNA-seq assays relevant in the sense of different stages of *Leishmania* to obtain transcriptional evidence to validate these predictions. The results revealed a majority of miRNA-like, as reported in *L. major,* most possess a length varying around 20–26 nt [47]. Given our results showing heterogeneity of size and number of predicted ncRNA observed in all species, we identified a median size of 23 nt for all these putative ncRNAs. This finding contrasts greatly with the data presented by Ruy and colleagues (2019), who observed a median size of 281 nt in their search in *L. braziliensis*. Interestingly, when comparing our method with other approaches, we realized that their rationale was based on the identification of non-coding transcripts and then asked for an RNA class. Instead, we based our search on sequence alignment and probabilistic models and then verified our findings using RNA-seq data. Ruy colleagues (2019) also found more lncRNAs than we did with our approach and our results showed a larger proportion of small ncRNAs [53]. This may suggest that they were identifying primary transcripts that could be precursors of small RNA classes identified in our analysis, which will require further analysis to be verified.

The conservation of ncRNA sequences has been observed in phylogenetically distant clades, such as between *Caenorhabditis elegans* and *Homo sapiens* [91], even in an inter-kingdom level, such as the miR485 family [92], which is conserved between *Arabidopsis thaliana*, *C. elegans*, *Mus musculus*, and *H. sapiens*; as well as in more closely related species, such as *A. thaliana* and *A. lyrata* [93]. In our case, considering the accepted phylogenetic relationship between *Leishmania* parasites and their potential to infect different hosts, as well as the ability of this pathogen to be associated with many clinical manifestations, we studied a putative relationship between sequence conservation of different ncRNAs classes and their role in parasite development. This was performed by comparing their expression profile in different developmental stages. The results we obtained here yield evidence of ncRNAs great conservation in *Leishmania* parasites among different species. These 876 core clusters (230,788 ncRNAs) represent more than 40% of all predicted ncRNAs. Additionally, as expected, we observed that conservation increases as phylogenetic distance decreases. Our analysis also identified an increase in ncRNA conservation in species that are canonically related to the same disease pathology or subgenus, as previously reported for *Leishmania* parasites [48,53] and other trypanosomatids [94]. Sequence conservation is also an indicator of selective pressure, as occurs with other species, and it can be associated with shared biological processes within related organisms [95]. Previous studies on other pathogens showed that many ncRNA classes are related to different developmental stages [96,97]. Thus, we observed that ncRNA conservation may indicate an important role in *Leishmania* development and in the physiopathology of leishmaniases.

To understand the differences in gene expression along distinct developmental stages in *Leishmania* parasites, we selected three representative strains according to their disease type, subgenus, and availability of RNA-seq datasets in public databases. In this sense, we compared the expression of both coding genes and ncRNAs from *L.* (*Viannia*) *braziliensis*, which causes cutaneous and mucocutaneous leishmaniasis; *L.* (*Leishmania*) *donovani*, which causes visceral leishmaniasis; and *L.* (*L.*) *major*, that causes cutaneous leishmaniasis. Our results show an expression of several proteins involved in host-parasite interaction in *L. braziliensis* amastigotes, such as major surface protease GP63 (leishmanolysin), a protein that is involved in the survival of intracellular amastigotes [98]. Also, this protein allows promastigotes to evade the complement-mediated lysis before its internalization by macrophages [99]. The expression of this gene was also observed in metacyclic promastigotes in *L. braziliensis*. Additionally, we also identified Biopterin Transporter (BT1) overexpression in this stage, which product plays a key role in growth, infectivity, and survival in the macrophages [100]. In two computational studies, the potential of the GP63 protein from *L. major* and *L. donovani* as a vaccine target was demonstrated [101,102]. Another study performed by Chowdhury et al. (2019), designed two siRNAs and three miRNAs that had *L. donovani* GP63 as their exclusive target, demonstrating that these molecules can be used to inhibit the expression of GP63 and act as one more therapeutic tool for tackling leishmaniasis [103]. This could inspire more tests to be performed on GP63 and to observe its potential as a vaccine and therapeutic target [104].

We identified the association of three ncRNA genes with the amastigote and procyclic developmental stages in *L. braziliensis*, based on a coding/non-coding gene coexpression network. Previously this approach has been employed to determine the function of different classes of ncRNAs, such as lncRNAs and miRNAs, in distinct etiological agents of infectious diseases, such as *Plasmodium falciparum* [83], *Toxoplasma gondii* [105], and *Schistosoma* [106,107].

We identified biological processes associated with the amastigote stage such as response to osmotic stress, hypotonic response and detection of osmotic stimulus in *L. braziliensis*, that involved one specific ncRNAs classified as lncRNA. Notably, the lncRNA (ncRNA_00056_lncRNA), shows significant coexpression with a putative gene for Amino acid permease (LbrM.10.0840), a vesicle-associated membrane protein (LbrM.27.2560) and a putative gene for aquaglyceroporin (LbrM.31.0020). In this context, Amino acid permease genes are required for *Leishmania* species for amino acid uptake, such as amino acid permease 3 (APP3) required for selective uptake of L-arginine [108]. Indeed, aquaglyceroporin are proteins that allow the transport of water, glycerol, and other small, uncharged solutes and plays an important role in osmoregulation [109,110]. The observed co-expression pattern for the lncRNA suggests it as a player in a possible regulatory role in modulating nutrient uptake, osmotic stress responses, and membrane dynamics during the amastigote stage.

In procyclic stage we found a sRNA (ncRNA14305_sRNA) significant coexpressed with a gene set that resulted in biological process enrichment related to pathogen motility, such as cytoskeleton organization, microtubule-based process, microtubule organizing center organization, ciliary basal body organization among others. In this sense, we can highlight the presence of alpha tubulin (LbrM.13.0210) and kinetoplastid membrane protein-11 (KMP-11) (LbrM.34.2150 and LbrM.34.2160) as coexpressed genes with the sRNA, suggesting a potential implication of this sRNA in cytoskeletal dynamics. Interestingly, KMP-11 is one of the major structural components of the surface membrane of *Leishmania* parasites and has been implicated in regulating the overall lipid bilayer morphology of the parasite membrane [111,112]. In fact, recent findings have revealed that KMP-11 facilitates the initial step of *Leishmania donovani* infection by modulating cholesterol transport and membrane fluidity, thereby promoting host cell invasion [112]. Briefly, KMP-11 forms oligomers that bridge the parasite and host macrophage membranes. This interaction is critically dependent on cholesterol (CHOL) and ergosterol (ERG) levels in the respective membranes. KMP-11 facilitates the transfer of cholesterol from the host macrophage to the parasite, which is essential for successful invasion [112]. Our findings, the co-expression of an sRNA with KMP-11 in the procyclic stage could suggest a regulatory mechanism, in which the sRNA may influence the expression or functional modulation of KMP-11. Given that membrane dynamics and fluidity are essential for the differentiation and survival of Leishmania within the vector, this sRNA might play a role in preparing the parasite for efficient host invasion.

In other hand, an unclassified ncRNA (ncRNA05461_Unclassified) was found to be coexpressed with Heat Shock Proteins HSP83 (LbrM.33.0350, LbrM.33.0340, LbrM.33.0330) and HSP110 (LbrM.18.1400). The protein set is involved in counteract stress conditions like high temperature, low pH, oxidative stress, change in nutritional availability and host inflammatory response through the folding, assembly, secretion, and the regulation of other protein [113]. In this sense, the co-expression results that involve the HSP set in *Leishmania* suggests a potential regulatory role of the ncRNA05461_Unclassified on the stress response during the procyclic stage. Given that the parasite must withstand environmental stressors within the sandfly vector and prepare for the transition to the mammalian host, the ncRNA05461_Unclassified might contribute to the fine-tuning of HSP expression.

In summary, our work is the first to identify novel ncRNAs in 25 genome isolates from 16 *Leishmania* species depicted from its pan-RNAome. We revealed a new set of non-coding genes that are involved in different developmental stages in the parasite life cycle and may play an important role in infection and survival. Furthermore, we obtained the co-expression profile of coding and non-coding genes functional insights for the potential role of a lncRNA and sRNA through guilty by association inferences. Importantly, our results provide novel evidence of possible mechanisms underlying co-regulation between coding-ncRNA, opening the way for further research on the role of these ncRNAs and their putative relationship with parasite survival.

## Supporting information

S1 Table Genome information of *Leishmania* species, and BUSCO completeness analysis. (XLSX)

S2 Table Publicly available data for *Leishamania* spp. The information corresponds to that reported directly on NCBI’s SRA platform. (XLSX)

S3 Table Non-coding RNA prediction and GC content compared vs genome sequence. (XLSX)

S4 Table Number of all classes of non-coding RNAs found in the genomes of *Leishmania* spp. (XLSX)

S5 Table Gene Ontology (GO) enrichment analysis of Biological Processes of Procyclic promastigotes DEG. (XLSX)

S6 Table Gene Ontology (GO) enrichment analysis of Biological Processes of Procyclic promastigotes DEG. (XLSX)

S7 Table Number of coding genes and ncRNAs per module in different *Leishmania* parasites. (XLSX)

S8 Table Biological processes enriched in Module related to Amastigote developmental stage in *L. braziliensis.* (XLSX)

S9 Table Biological processes enriched in Module related to Metacyclic promastigote developmental stage in *L. braziliensis.* (XLSX)

S10 Table Biological processes enriched in Module related to Procyclic promastigote developmental stage in *L. braziliensis.* (XLSX)

S11 Table Biological processes of hub genes in Amastigote developmental stage in *L. braziliensis.* (XLSX)

S12 Table Biological processes of hub genes in Procyclic promastigote developmental stage in *L. braziliensis.* (XLSX)

S1 Fig Module associated to Amastigote developmental stage in *L. braziliensis. In orange represented the ncRNAs and in purple the protein-coding genes.* (DOCX)

S2 Fig Module associated to Procyclic promastigote developmental stage in *L. braziliensis.* In orange represented the ncRNAs and in purple the protein-coding genes. (DOCX)

S3 Fig Module associated to Metacyclic promastigote developmental stage in *L. braziliensis.* In orange represented the ncRNAs and in purple the protein-coding genes. (DOCX)
