# Supplementary material for: Comparative and systems analyses of Leishmania spp. non-coding RNAs through developmental stages
Source: PLoS Negl Trop Dis. 2025 May 28;19(5):e0013108. doi: 10.1371/journal.pntd.0013108 (PMC12169548; doi:10.1371/journal.pntd.0013108)
Supplement: S1 Fig — (DOCX) [file pntd.0013108.s013.docx]

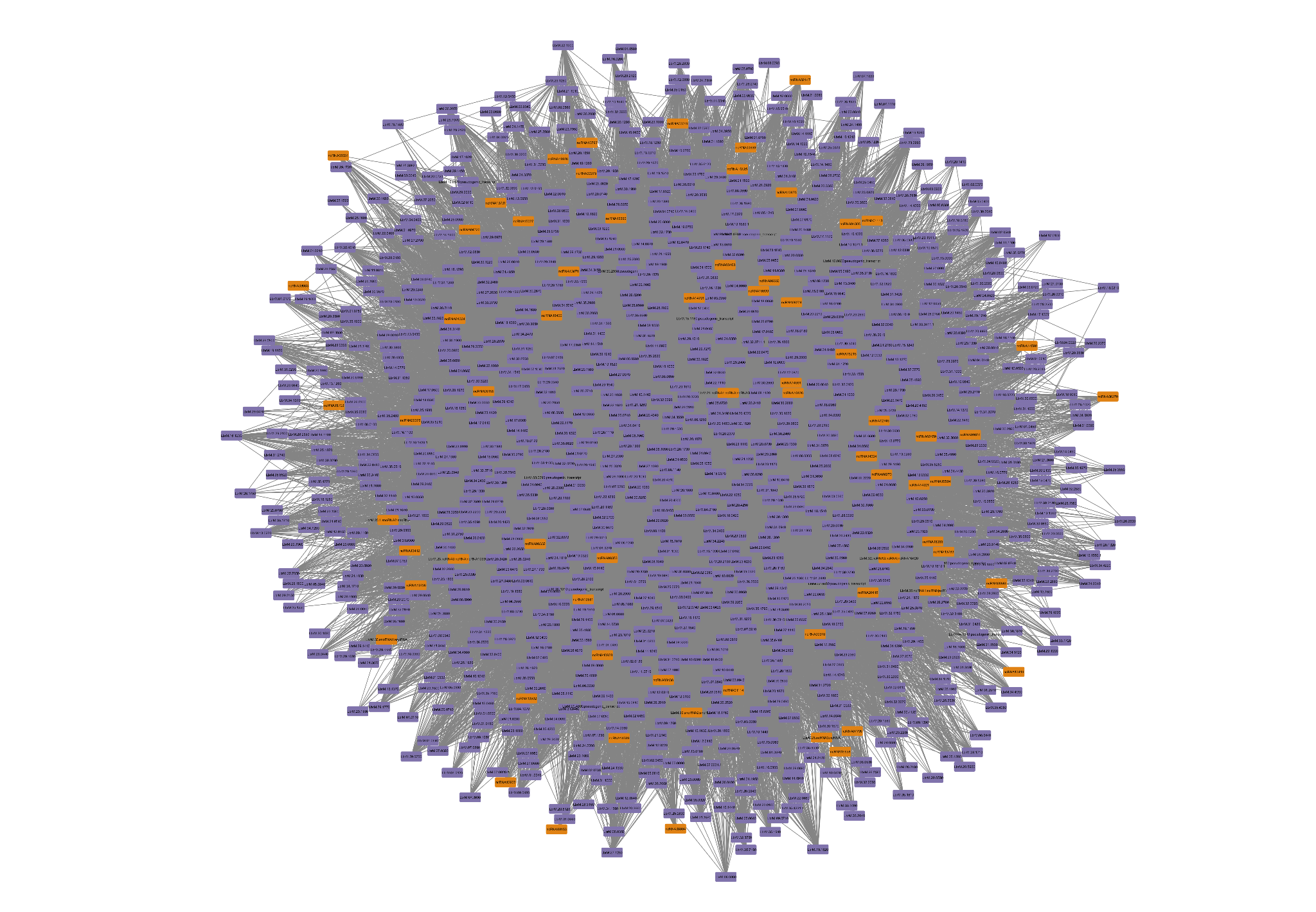


Supplementary Figure S1. Module associated to Amastigote developmental stage in *L. braziliensis*. In orange represented the ncRNAs and in purple the protein-coding genes
