# Supplementary material for: Comparative and systems analyses of Leishmania spp. non-coding RNAs through developmental stages
Source: PLoS Negl Trop Dis. 2025 May 28;19(5):e0013108. doi: 10.1371/journal.pntd.0013108 (PMC12169548; doi:10.1371/journal.pntd.0013108)
Supplement: Attachment — Submitted filename: rebuttal_letter_PlosNTD.pdf [file pntd.0013108.s017.pdf]

Dear Editors,

We sincerely appreciate the opportunity to revise and resubmit our manuscript titled "**Comparative and systems analyses of *Leishmania* spp. non-coding RNAs through developmental stages**". We are grateful for the constructive feedback from the reviewers, which has helped us improve the quality and clarity of our study. Below, we provide a detailed point-by-point response to each comment. Changes made in the manuscript have been clearly indicated.

Journal Requirements:

1) Please provide an Author Summary. This should appear in your manuscript between the Abstract (if applicable) and the Introduction, and should be 150 200 words long. The aim should be to make your findings accessible to a wide audience that includes both scientists and non-scientists. Sample summaries can be found on our website under Submission Guidelines:

A: We have included an Author Summary section between the Abstract and Introduction in the manuscript, following the journal's guidelines.

2) We have noticed that there is a reference to supplementary figure 4;however, there is no corresponding file uploaded to the submission. Please upload it as a separate file with the item type 'Supporting Information'.

A: We have uploaded the missing supplementary figure as a separate file under "Supporting Information."

3) We have noticed that you have uploaded Supporting Information files, but you have not included a list of legends. Please add a full list of legends for your Supporting Information files after the references list.

A: We have added a complete list of legends for all Supporting Information files after the reference section.

4) Please amend your detailed Financial Disclosure statement. This is published with the article. It must therefore be completed in full sentences and contain the exact wording you wish to be published.

> 1) State the initials, alongside each funding source, of each author to receive each grant. For example: "This work was supported by the National Institutes of Health (####### to AM; ###### to CJ) and the National Science Foundation (###### to AM)."
> 2) State what role the funders took in the study. If the funders had no role in your study, please state: "The funders had no role in study design, data collection and analysis, decision to publish, or preparation of the manuscript.".
> If you did not receive any funding for this study, please simply state: The authors received no specific funding for this work.

A: We have revised the Financial Disclosure section to clearly state the funding sources, including author initials, and confirmed that the funders had no role in study design, data collection, analysis, decision to publish, or manuscript preparation.

5) Please ensure that the funders and grant numbers match between the Financial Disclosure field and the Funding Information tab in your submission form. Note that the funders must be provided in the same order in both places as well. Currently," the supercomputing infrastructure of the NLHPC (ECM- 02)" is missing from the Funding Information tab while grant number "Financiamiento Basal para Centros Científicos y Tecnológicos de Excelencia de ANID: Funadación Ciencia & VidaFB210008" is missing from the Financial Disclosure field. Please ensure that the order of the funders exactly match in the Financial Disclosure field and the Funding Information tab.

A: We have ensured that all funders and grant numbers match between the Financial Disclosure field and the Funding Information tab, maintaining the same order.

6) Thank you for providing us with the Data Availability statement. Please provide us with direct links to access the datasets.
A: We have provided direct links to access the datasets in the Data Availability section.

Reviewers' Comments:

Reviewer #1:

The main objective of this study was to determine the non-coding RNAs (ncRNAs) repertoire in several Leishmania species. For this purpose, the authors used publicly available data consisting of genomic sequences and RNA-seq Illumina reads. The authors applied several bioinformatics tools and generated many results. However, the design of the study and methodological details need to be better explained; otherwise, the relevance of the results would be questionable.

Main points.

1) Clarification on how the authors predict the repertoire of ncRNAs (section 2.2.) is mandatory. Reference 58 describes a database created by compilation of non-coding RNA (ncRNAs) classes described in organisms of different kingdoms. Questions:

i) How ncRNAs were identified (Blast, structural features, etc.)?

A: We have expanded Section 2.2 of the Methods to provide a clearer and more comprehensive description of the steps taken, tools used, and parameters applied for the prediction of non-coding RNAs (ncRNAs). Briefly, ncRNA identification was performed using a dual approach: i) **Covariance models** to capture the structural features of known RNA families, and ii) **Sequence homology searches**. For the latter, we used **Bowtie2** to map potential ncRNAs with an approximate **80% sequence similarity** to entries in the NR2 database, specifically filtered to avoid non-eukaryotic non-coding RNAs (such as bacterial).

Additionally, to validate the expression of the predicted ncRNAs, we incorporated **transcriptional evidence from RNA-seq data** obtained under various conditions, enabling us to assess their expression across different developmental stages of *Leishmania* spp. Based on this evidence, we can confidently assert that the identified ncRNAs are real RNA molecules that are actively transcribed. By integrating structural predictions with expression

data from public databases, we ensured a **robust annotation** of the ncRNAs identified in this study.

ii) Do the authors know if the identified ncRNAs are real RNA molecules in Leishmania?

A: In Section 2.3 *Transcriptional evidence of predicted ncRNAs* , we elaborate on the approach used to assess transcriptional evidence, specifically through FPKM normalization, which was utilized as a criterion to determine the existence of predicted ncRNAs.

2) Figure 1 and accompanying text in the manuscript. Apart from rRNAs and tRNAs, what were the structural features used to define each category of ncRNAs? Most of the ncRNAs are within the groups “unclassified” and “others”. However, how the authors deduce they are ncRNAs is unclear. Without a clear explanation, their existence might be questionable.

A: Thank you for your comment. Based on this feedback, we have reviewed the prediction and classification of ncRNAs in *Leishmania* genomes. To refine our annotations, we applied a weighted approach to the classification process using a Python script that systematically evaluated all possible combinations of functional annotations assigned to the predicted ncRNAs. The specific annotations guidelines are documented in the file *clases_ncRNA_final.txt*, which has been deposited in our GitHub repository.

The classification criteria were established as follows:

1. If an ncRNA was annotated by Rfam, its functional annotation was automatically assigned, irrespective of any additional annotations provided by NR2 or sequence similarity to previously predicted ncRNAs in *Leishmania*.
2. If an ncRNA was not annotated by Rfam but exhibited sequence similarity to a previously predicted ncRNA in *Leishmania*, the corresponding functional annotation was transferred.
3. If an ncRNA lacked annotations based on Rfam or sequence similarity to previously described ncRNAs in *Leishmania*, we considered the annotations provided by NR2.
4. Additionally, in cases where an ncRNA had multiple distinct functional annotations (e.g., sRNA, siRNA, miRNA, or piwiRNA), it was classified as sRNA (small RNA). ncRNAs shorter than 200 nucleotides were also categorized as sRNA.
5. An ncRNA was classified as "unclassified" if it exclusively carried an NR2 annotation without any additional functional assignment or if there were ambiguities in the Rfam predictions. For instance, if an ncRNA was simultaneously annotated as both a miRNA and a cis-regulatory RNA, it was considered ambiguous and subsequently classified as "unclassified."

3) In the legend to figure 1, it is read: ncRNAs gene prediction was validated by transcriptional evidence using RNA-seq analysis. What the evidence was (the existence of RNA-seq reads or the existence of annotated transcripts)? This is another relevant question to be clarified. The sole existence of reads is not a demonstration that the predicted ncRNAs exist such as annotated in this study.

A: We appreciate the reviewer’s insightful comment. The RNA-seq analysis provided **transcriptional evidence** supporting the presence of the predicted ncRNAs. Specifically, we identified **mapped reads that overlapped with the predicted ncRNA loci**, consistent with their expression. While the presence of RNA-seq reads alone does not definitively prove the exact annotation of the ncRNAs, it strongly indicates that these regions are **transcriptionally active** and likely correspond to **real ncRNA molecules**.

Furthermore, by integrating **covariance model predictions** and **sequence homology-based annotations** with this RNA-seq evidence, we enhanced the confidence that these predicted ncRNAs are indeed transcribed and biologically relevant. This combined approach provides a more robust validation of the ncRNA predictions made in this study.

4) Line 222. Please, specify the difference between dispensable ncRNAs and accessory-RNAome.

A: To avoid potential confusion, we acknowledge that there is no significant distinction between the two terms. Therefore, we have decided to use only the term **Accessory RNAome** throughout the manuscript for consistency.

5) Line 242. It is read: These Viannia-specific clusters were primarily annotated as gene expression regulatory ncRNAs. What criteria served to define "gene expression regulatory ncRNAs"? Please, comment on it.

A: Using the revised functional annotation, we have clarified in the article this aspect to ensure greater accuracy and consistency in the classification of ncRNAs.

6) Lines 364-365. What is the meaning of "the LbrM2903_20.1_984445-984477_miRNA miRNA-like gene was co-expressed with 40 coding genes"? How co-expression was determined?

A: We employed a well-established methodology, widely described in various studies, for the characterization of potential ncRNA functions through co-expression networks. Specifically, we utilized the Weighted Gene Co-expression Network Analysis (WGCNA) R package, which constructs a scale-free network based on pairwise correlations of gene expression levels. This approach enables the identification of gene modules composed of genes with highly correlated expression patterns. For a more detailed explanation of the cutoffs and parameters used in this analysis, please refer to Section 2.5: *Co-expression Network Analysis* in the Methods.

Furthermore, the observation that the ncRNA *LbrM2903_20.1_984445-984477_miRNA miRNA-like* co-expresses with 40 coding genes indicates that its expression pattern is highly similar to that of these genes under the experimental conditions tested, specifically in *L. braziliensis* amastigotes.

7) Although the manuscript includes a quite large list of references, there are relevant articles missed by the authors. Examples:

- Freitas Castro, F., Ruy, P. C., Nogueira Zeviani, K., Freitas Santos, R., Simoes Toledo, J. and Kaysel Cruz, A. (2017). Evidence of putative non-coding RNAs from

Leishmania untranslated regions. Mol Biochem Parasitol 214, 69–74. doi: 10.1016/j.molbiopara.2017.04.002.

- Torres, F., Arias-Carrasco, R., Caris-Maldonado, J. C., Barral, A., Maracaja-Coutinho, V. and De Queiroz, A. T. L. (2017). LeishDB: a database of coding gene annotation and non-coding RNAs in Leishmania braziliensis. Database (Oxford) 2017, bax047. doi: 10.1093/database/bax047.

Ruy, P. D. C., Monteiro-Teles, N. M., Miserani Magalhaes, R. D., Freitas-Castro, F., Dias, L., Aquino Defina, T. P., ... & Kaysel Cruz, A. (2019). Comparative transcriptomics in Leishmania braziliensis: disclosing differential gene expression of coding and putative noncoding RNAs across developmental stages. RNA biology, 16(5), 639-660.

Detailed comparisons between the data reported in these studies (and others cited in the manuscript) and the results generated in this study merit being carried out.

A: We have incorporated the suggested references and included a comparison of our data with previous studies on Leishmania ncRNAs.

Reviewer #2:

1) Please include a table or supplementary material listing all genomes used, including genome size, annotation information, database IDs, and a completeness measure, such as BUSCO scores.

A: We have added in the supplementary table 1 the list of genome sizes, annotation information, database IDs, and BUSCO scores.

2) To enhance transparency, provide a supplementary table detailing the RNA-seq data used, including species, strains, replicates, number of reads, and sequencing technology.

A: We have included in *Supplementary Table 2* a comprehensive list detailing the RNA-seq data used in this study, including information on species, strains, number of replicates, read counts, and sequencing technology.

3) Improve the explanation of the two approaches for ncRNA prediction. For the first approach, the database link is missing, and the scope of the database used is unclear. Specify the database size. Additionally, the tools and parameters used for the search are not well-documented and are not available in the GitHub repository (ncRNA_leish). For the second approach, clarify how each tool was applied and the specific settings used.

A: The parameters for all software used in this study, along with the filtering criteria applied for expression validation, have been explicitly detailed in the Methods and Results sections.

4) The methodology for validating ncRNA predictions is not sufficiently detailed. Explicitly describe the parameters rather than referencing other publications. Provide parameters for tools such as Bowtie and HTSeqCount. Additionally, mention the CPM filter used in the results section.

A: The methods and parameters used for validating ncRNA predictions were rewritten and described in greater detail, the process in *Section 2.3: Transcriptional Evidence of Predicted ncRNAs* in the Methods.

5) Comparative analysis of ncRNAs: On line130, specify the coverage used. The Gblocks parameters also need to be detailed.

A: Coverage used in sequence comparison has been specified, and Gblocks parameters are now detailed.

6) The characterization of RNAi/miRNA should be reorganized, possibly by including subsections within the methodology for different genomes.

A: Following the suggestion of Reviewer 3, we have removed the section *"Identification of Putative RNA Interference Pathway in Leishmania Genomes"* from our manuscript, as it does not align with the main focus of the study.

7) When identifying AGO1-like proteins, the authors used BLAST, but could an HMM-based search provide complementary insights?.

A: Following the suggestion of Reviewer 3, we have removed the section *"Identification of Putative RNA Interference Pathway in Leishmania Genomes"* from our manuscript, as it does not align with the main focus of the study.

8) The abundance of detected ncRNA families could be influenced by genome completeness or size. Consider discussing this in more detail.
A: Based on the BUSCO completeness analysis, we decided to exclude the genome of *L. peruviana* LEM1537 from our study. However, we did not find a direct relationship between genome completeness and the number of predicted ncRNAs. This suggests that the observed differences may be influenced by other factors, such as the number of contigs. Nevertheless, when evaluating the predictions, this correlation was not particularly strong either, indicating that additional factors might be contributing to the observed variation in ncRNA abundance.

9) The distribution of clusters generated by CD-HIT and analyzed in the RNAome is not fully clear. For example, 7.95% represents 196,000 ncRNAs, while 92.05% represents 76,000 ncRNAs. Are these dispensable ncRNAs supported by transcriptomic data? And 10) A notable cluster is absent in L. sp. D974 in Figure 2. Could this be due to genome annotation or completeness issues?

A: To mitigate potential biases in the conservation analysis of ncRNAs across *Leishmania* species, we refined our ncRNA prediction approach. This improvement involved optimizing the filtering criteria and incorporating additional comparative analyses to enhance the accuracy and reliability of predicted ncRNAs. These adjustments allowed for a more robust assessment of ncRNA conservation across species, reducing the influence of potential artifacts related to genome assembly quality or annotation inconsistencies.

11) l.236: Correct the phrase “In terms.”

A: Corrected in the manuscript.

12) l.257–266: This section would benefit from reorganization. First, describe the total number of DEG genes identified in each species. Then focus on L. braziliensis, highlighting common DEGs between stages and associated GO terms. Finally, present results from the other two species in a separate paragraph.

A: We have implemented the recommendations of Reviewer 3 by utilizing the AmiGO2 software to reorganize the hierarchy of the GO enrichment analyses, ensuring a more structured and accurate representation of the results.

13) Lines 281–305: Improve the hierarchy of the Gene Ontology analysis similarly to the suggestion above.

A: We have implemented the recommendations of Reviewer 3 by utilizing the AmiGO2 software to reorganize the hierarchy of the GO enrichment analyses, ensuring a more structured and accurate representation of the results.

14) Line 311: Clarify "In contrast."

A: Corrected in the manuscript

15) Figure 5: Consider a different representation of co-expression networks. The current labels (IDs) are unreadable. A simplified network diagram with circular nodes in two colors (without labels) could visually illustrate the differences in the number of coding genes and miRNA-like transcripts between species and improve the aesthetics of the figure.

A: Reviewer 2's suggestion regarding the visualization of co-expression networks was taken into account, and the corresponding modifications are reflected in *Figures 5* and *6*.

16) Figure 6: Similarly, parts A, B, and C in this figure are not informative in their current form. Consider alternative visualizations.

A: Reviewer 2's suggestion regarding the visualization of co-expression networks was taken into account, and the corresponding modifications are reflected in *Figures 5* and *6*.

17) Line 407: Please revise the phrasing for clarity.

A: Corrected in the manuscript

18) In the phylogeny presented in Fig.suppl. 1, some nodes can be rotated to better show the association between the phylogeny and the dendogram. Improved this figure is even interesting to show as the main figure.

A: *Supplementary Figure 1* was revised and improved based on the reviewer's suggestion and has now been incorporated into *Figure 2* of the main manuscript.

19) l.53: Replace "macrophage" with "cells (neutrophils, macrophages, and dendritic cells)."

A: Corrected in the manuscript.

20) I.88: The phrase "either conserved or unique among these species" seems redundant. Could the authors clarify the intended point?

A: Corrected in the manuscript

21) This manuscript explores the role of non-coding RNAs (ncRNAs) in the development and pathogenesis of Leishmania spp., the protozoan parasites responsible for leishmaniasis. The study characterizes the ncRNA repertoire across 26 strains from 16 different Leishmania species, identifying both conserved and unique ncRNA clusters. It also analyzes expression patterns in relation to coding genes, revealing the significant involvement of ncRNAs in regulating parasite development and survival. Notably, several miRNA-like transcripts were found to be co-expressed with coding genes associated with starvation, survival, and histone modification. Additionally, the authors investigate the presence of an RNA interference (RNAi) pathway in Leishmania, identifying some components of the pathway while suggesting the possibility of a non-canonical RNAi mechanism due to the absence of key canonical components.
Despite its significant contribution to the understanding of ncRNAs—particularly miRNA-like RNAs—and the potential identification of non-canonical RNAi pathways, the article could benefit from improvements in the methodological rigor and a more structured and hierarchical presentation of the results. Specifically, the quality and completeness of the datasets used for inference are not sufficiently discussed.
Overall, the manuscript is well-presented; however, given its focus on a comprehensive in silico analysis, it would benefit from stronger support in terms of data quality and applied methodologies. The presentation of a large amount of data and comparisons makes the narrative difficult to follow at times. A clearer hierarchical structure within sections and the use of shorter sentences would improve the readability and coherence of the results.

A: We appreciate the reviewer's positive remarks on the significance of our study. To enhance readability and improve methodological rigor, we have structured the Results section more hierarchically, ensuring a logical flow from ncRNA identification to functional analysis.

We have revised the text to clarify the completeness and quality control of the datasets used, explicitly describing the genome completeness assessment (BUSCO scores) and quality filtering of RNA-seq data.

We have reduced sentence complexity and improved narrative coherence throughout the manuscript, making it more accessible for the reader.

Reviewer #3:

1) In the first section "Dissecting the repertoire of non-coding RNAs across Leishmania spp." the authors used computational combined approaches that includes covariance model comparisons of known RNA families, sequence similarity searches from public databases, and RNA-sequencing datasets to map the ncRNA repertoire across 26 genomes from 16 Leishmania species.
The analysis was based in two databases, NRDR/NR2 and Rfam v14, and the proportion of miRNA, siRNA and piRNA seems to be too high and too homogeneous across Leishmania species (Fig1A). There seems to be a bias that enriches the

classes linked to the RNAi pathway, which only exists in the Viannia subgenus. It seems to this reviewer that the analysis may be biased by the databases used. I consider necessary that the authors use the annotation already available for well-known structural ncRNAs, such as rRNA and tRNA, in the genome of L. major (best annotated genome) to check for the proportion of these families to confirm their prediction.

A: We have validated our predictions using *L. major* annotations for ncRNAs described in the TritrypDB, an online database of genomic data for kinetoplastid parasites, such as *Leishmania* and *Trypanosoma*. and a dataset of siRNAs sequencing from *L. braziliensis* (accession SRR582267, https://www.ncbi.nlm.nih.gov/), to ensure accuracy.

2) Data presented in Fig1C is confusing. The authors used different scales in each panel making it difficult to compare, and the scales used do not help, for the few tick markers. Also, it calls the attention that in all families presented, the L. braziliensis levels are always lower even in the miRNA and piRNA families, which seems to be a contradiction, reinforcing the strangeness based on what is known about this machinery in Leishmania. It is also unexpected the low rRNA abundance, I guess that this would be true if the transcriptomes used as the source of the analysis were rRNA depleted. But the authors fail to indicate that and the presentation of levels of rRNAs using a rRNA depleted library, for their purposes, makes no biological sense and is misleading.

A: We have standardized scales and improved clarity for comparative analysis.

3) Despite the putative 'RNA families' bias inserted by the first steps of the computational approaches (as abovementioned) to identify represented RNA families, data analysis and corresponding representation in the section "The Leishmania spp. pan-RNAome: ncRNAs conservation across 26 genomes" is novel and brings information on sequence conservation of the putative ncRNAs with an interesting hierarchy of core/accessory/unique transcripts identified. In the lines 249-255 the considerations made on the phylogenetic/clusterization results are noteworthy as this analysis may be indicative of similar evolutionary patterns for coding and non-protein coding genes, which could have been further explored. Moreover, the authors mention the data 'en passant' in the discussion and it seems to this reviewer that these data deserve a more thorough discussion. It would be worthy for the authors to consider highlighting the data in the discussion.

A:

We sincerely appreciate the reviewer's feedback regarding the phylogenetic and clustering analyses presented in the section *"The Leishmania spp. pan-RNAome: ncRNAs conservation across 26 genomes."* We acknowledge that the evolutionary considerations related to the **hierarchy of core, accessory, and unique transcripts**, as well as the observed similarities in clustering patterns between coding and non-coding genes, were addressed only briefly in the original discussion. In response to this valuable suggestion, we provide a more thorough interpretation of these findings.

4) In the section "Stage-specific differential expression of coding genes and ncRNAs" it is very difficult for the reader to follow the information presented in Figure 3 and accompanying text. Difficult to figure out the numbers presented, the text is not clear, and figure legend doesn't help. The figure and text must be profoundly modified. In addition, authors inform that they have analysed DEG in L. donovani, L. major and L. braziliensis. L. donovani and L. major are presented in the supplementary file but in a distinct form, making any comparative analysis troublesome. The reader must go to each figure to understand and compare the

numbers presented. The L. braziliensis data (Fig3A) presentation is very confusing.
Besides, the GO analysis presented lacks important information; the parent/children relationship of the terms is not discussed, inserting a bias to the figure as presented. In addition, p-values indicate that many of the GO enrichments are not soundly supported by the statistics. In the case of the metacyclics, none of the GOs pointed as enriched have a sound statistics support, and the number of genes per GO is very low. None of this is discussed or carefully presented. We suggest the authors to use a tool to make the parent/children relationship clear (AmiGO 2 > Tools & Resources > Visualization > GO IDs or advanced format (Paste json file) > Use advanced format > Output type: SVG > Visualize) and to conduct the reader on the significance of data as presented.

A: We have implemented the recommendations of Reviewer 3 by utilizing the AmiGO2 software to reorganize the hierarchy of the GO enrichment analyses, ensuring a more structured and accurate representation of the results.

5) The section “Co-expression network analysis identifies developmental stages-associated gene modules in Leishmania spp.” needs major adjustments:
5.1. This reviewer couldn’t understand how the network was constructed and how the modules were associated with each stage, the methodology must be better explained (WGCNA and GS and MM Scores used to determine correlations must be included in the methodology). It is possible that I did not understand because I do not work with network construction, but this will be true for most of the readers. Furthermore, the construction of networks and correlations are central to the next analyses conducted, so it is essential that these aspects are clearly presented.

A: We employed a well-established methodology, widely described in various studies, for the characterization of potential ncRNA functions through co-expression networks. Specifically, we utilized the Weighted Gene Co-expression Network Analysis (WGCNA) R package, which constructs a scale-free network based on pairwise correlations of gene expression levels. This approach enables the identification of gene modules composed of genes with highly correlated expression patterns. For a more detailed explanation of the cutoffs and parameters used in this analysis, please refer to Section 2.5: *Co-expression Network Analysis* in the Methods

6) 5.2. Supplementary Fig 4 was not included, thus, we have only the L. braziliensis heatmap. This reviewer considers that after explaining rational and approaches for these results, the heatmaps (L. braziliensis, L. donovani and L. major) should be all part of supplementary material.

A: Corrected in the manuscript

6) When we get to the section “Detection of potential miRNA-like involved in amastigote life cycle in Leishmania parasites”, the authors start (line 337) by stating that To assess the possible functions of the identified miRNA-like in amastigote developmental stages, DE mRNAs and miRNAs-like RNAs were selected to filter the co-expression network corresponding to this life cycle stage in L.braziliensis and L. donovani.
The idea of filtering the co-expression network in a specific life-stage is interesting as with the filtering authors would avoid the difficulties of GO terms. Nevertheless, the filtering procedure is not clearly explained; the source of the numbers of DE mRNAs from L. braziliensis and L. donovani are not well-defined. What was the source used to reach 89 and 206 amastigotes preferentially expressed genes (mRNAs) in L. braziliensis and L. donovani? The numbers do not match with numbers from Fig 3 and if they come from articles published

it must be indicated. Also, authors state (line 344) that 206 protein coding genes are exclusively expressed in amastigotes which is a very strong statement, given that few genes are known to be ‘exclusive’ of a given stage in these parasites, most of the DEGs in Leishmania are preferentially expressed but not “exclusive” of a given life stage.
Next, the authors apply a further filtering step (line 363) - Through the guilty by association functional annotation approach, we predicted the possible function of the top miRNA like that participates in the amastigote developmental stage in L. braziliensis and L. donovani. - with another methodology which is not even referred in the methods section.
Also, to the GO enrichment analyses presented in Figures 5 and 6 this reviewer extends the critique raised over similar data shown in Fig 3. As mentioned above: the parent/children relationship of the terms are not discussed, inserting a bias to the figure as presented. In addition, p-values indicate that many of the GO enrichments are not soundly supported by the statistics.

A: We have clarified the filtering procedure for selecting differentially expressed (DE) mRNAs and miRNA-like RNAs in L. braziliensis and L. donovani. The source of the numbers (89 and 206 DE mRNAs) has been explicitly stated in the Methods section.
The term "exclusively expressed" has been corrected to "preferentially expressed" to reflect a more accurate biological interpretation.
The "guilty by association" annotation approach has now been fully detailed in the Methods section to ensure transparency.
Figures 5 and 6 have been revised to provide a clearer presentation of GO enrichment, addressing concerns regarding parent/child relationships and statistical significance

7) In the last results section “Identification of putative RNA interference pathway in Leishmania genomes” there is basically no novelties. That RNAi pathway is complete in Leishmania species of the subgenus Viannia is known since 2007 (reference 35, which is listed but not cited in the text). After discovery of the RNAi machinery in L. braziliensis, other authors explored functionally the pathway. Thus, it seems to this reviewer that the section and Figure 7 contains nothing that has not been explored and presented before. The authors should present in the results section only the new information highlighting what is new compared to what is publicly available.
In addition, Fig7D brings expression results that are misleading. For instance: the first panel of Fig7D presents AGO1 expression in L. major and L. donovani, but it is known that there are only remnants of AGO1 in Leishmania subgenus. So, what is the purpose of presenting the levels of expression of a gene known to be non-functional? What is the purpose of panel D? The form the authors present the data on expression of the other genes included in the same panel is also misleading; just to cite one, Exportin 1 is not an exclusive RNAi pathway protein. Exportin 1 has been studied by others and it has been shown, for instance, that it mediates nuclear export of SLRNA. So, what is the reason for the authors to present levels of expression of different genes with an unclear or unproved link to the RNAi pathway, which is, in addition, non-functional in the species of subgenus Leishmania?
Thus, in conclusion, this last topic should be either deleted or profoundly modified to show and highlight something new.

A: Following the suggestion of Reviewer 3, we have removed the section "Identification of Putative RNA Interference Pathway in Leishmania Genomes" from our manuscript, as it does not align with the main focus of the study.

8) Some of the conclusions are not supported by the data presented (as noted in the comments above). If the critiques raised regarding certain analyses, results, and data presentation are accepted, the conclusions and interpretations drawn from these findings will need to be revised. The authors should ensure that their conclusions align more closely with the data presented and address the potential biases and limitations discussed. Clearer justification and contextualization of their results are essential to strengthen the manuscript's overall validity and impact.

A: We have critically revised the conclusions to ensure they align strictly with the data presented. Potential biases and limitations have been explicitly discussed to strengthen the manuscript's validity and impact.

Additional justifications have been provided for key findings, and interpretations have been refined where necessary to avoid overstatement.

9) The authors aimed to identify ncRNAs from Leishmania spp. to gain insights into the RNAome (repertoire of ncRNAs) of this protozoan genus by analyzing a large number of strains and species. They performed computational analyses to investigate co-expression relationships between coding genes and ncRNAs expressed in all stages of Leishmania braziliensis, L. donovani and L. major. While the study is commendable for incorporating a substantial number of Leishmania strains and species (26 strains representing 16 different species) and different levels of supporting data, there are significant limitations in the work that currently prevent its recommendation for publication.

A: We acknowledge the reviewer's concerns regarding dataset limitations. To address this, we have expanded our discussion on the dataset completeness and methodological considerations, ensuring transparency in the limitations of our computational approach.
