# Supplementary material for: Comparative and systems analyses of Leishmania spp. non-coding RNAs through developmental stages
Source: PLoS Negl Trop Dis. 2025 May 28;19(5):e0013108. doi: 10.1371/journal.pntd.0013108 (PMC12169548; doi:10.1371/journal.pntd.0013108)
Supplement: Peer review history

# Author comment: Author response to Decision Letter 0

**DOI:** 10.1371/journal.pntd.0013108.r001

14 Oct 2024

# Peer review: Decision Letter 0

**DOI:** 10.1371/journal.pntd.0013108.r002

**Authors:** Claudia Brodskyn

21 Nov 2024

PNTD-D-24-01486Comparative and systems analyses of Leishmania spp. non-coding RNAs through developmental stagesPLOS Neglected Tropical Diseases Dear Dr. Martin, Thank you for submitting your manuscript to PLOS Neglected Tropical Diseases. After careful consideration, we feel that it has merit but does not fully meet PLOS Neglected Tropical Diseases's publication criteria as it currently stands. Therefore, we invite you to submit a revised version of the manuscript that addresses the points raised during the review process. Please submit your revised manuscript within 60 days Jan 20 2025 11:59PM. If you will need more time than this to complete your revisions, please reply to this message or contact the journal office at plosntds@plos.org. When you're ready to submit your revision, log on to https://www.editorialmanager.com/pntd/ and select the 'Submissions Needing Revision' folder to locate your manuscript file. Please include the following items when submitting your revised manuscript: * A rebuttal letter that responds to each point raised by the editor and reviewer(s). You should upload this letter as a separate file labeled 'Response to Reviewers '. This file does not need to include responses to any formatting updates and technical items listed in the 'Journal Requirements' section below. * A marked-up copy of your manuscript that highlights changes made to the original version. You should upload this as a separate file labeled 'Revised Manuscript with Track Changes '. * An unmarked version of your revised paper without tracked changes. You should upload this as a separate file labeled 'Manuscript '. If you would like to make changes to your financial disclosure, competing interests statement, or data availability statement, please make these updates within the submission form at the time of resubmission. Guidelines for resubmitting your figure files are available below the reviewer comments at the end of this letter. We look forward to receiving your revised manuscript. Kind regards,Claudia Ida BrodskynSection EditorPLOS Neglected Tropical Diseases Claudia BrodskynSection EditorPLOS Neglected Tropical Diseases

Shaden Kamhawi

co-Editor-in-Chief

PLOS Neglected Tropical Diseases

orcid.org/0000-0003-4304-636XX

Paul Brindley

co-Editor-in-Chief

PLOS Neglected Tropical Diseases

orcid.org/0000-0003-1765-0002

**Journal Requirements:**

1) Please provide an Author Summary. This should appear in your manuscript between the Abstract (if applicable) and the Introduction, and should be 150u2013200 words long. The aim should be to make your findings accessible to a wide audience that includes both scientists and non-scientists. Sample summaries can be found on our website under Submission Guidelines:

https://journals.plos.org/plosntds/s/submission-guidelines#loc-parts-of-a-submission

2) We have noticed that there is a reference to supplementary figure 4;however, there is no corresponding file uploaded to the submission. Please upload it as a separate file with the item type 'Supporting Information'.

3) We have noticed that you have uploaded Supporting Information files, but you have not included a list of legends. Please add a full list of legends for your Supporting Information files after the references list.

4) Please amend your detailed Financial Disclosure statement. This is published with the article. It must therefore be completed in full sentences and contain the exact wording you wish to be published.

1) State the initials, alongside each funding source, of each author to receive each grant. For example: "This work was supported by the National Institutes of Health (####### to AM; ###### to CJ) and the National Science Foundation (###### to AM)."

2) State what role the funders took in the study. If the funders had no role in your study, please state: "The funders had no role in study design, data collection and analysis, decision to publish, or preparation of the manuscript.".

If you did not receive any funding for this study, please simply state: u201cThe authors received no specific funding for this work.u201d

5)  Please ensure that the funders and grant numbers match between the Financial Disclosure field and the Funding Information tab in your submission form. Note that the funders must be provided in the same order in both places as well. Currently," the supercomputing infrastructure of the NLHPC (ECM- 02)" is missing from the Funding Information tab while grant number  "Financiamiento Basal para Centros Científicos y Tecnológicos de Excelencia de ANID: Funadación Ciencia & VidaFB210008" is missing from the Financial Disclosure field. Please ensure that the order of the funders exactly match in the Financial Disclosure field and the Funding Information tab.

6) Thank you for providing us with the Data Availability statement. Please provide us with direct links to access the datasets.

**Reviewers' Comments:**Reviewer's Responses to Questions

**Key Review Criteria Required for Acceptance?**

As you describe the new analyses required for acceptance, please consider the following:

**Methods**

-Are the objectives of the study clearly articulated with a clear testable hypothesis stated?

-Is the study design appropriate to address the stated objectives?

-Is the population clearly described and appropriate for the hypothesis being tested?

-Is the sample size sufficient to ensure adequate power to address the hypothesis being tested?

-Were correct statistical analysis used to support conclusions?

-Are there concerns about ethical or regulatory requirements being met?

Reviewer #1: -Are the objectives of the study clearly articulated with a clear testable hypothesis stated? NO

-Is the study design appropriate to address the stated objectives? NO

-Is the population clearly described and appropriate for the hypothesis being tested? NO

Reviewer #2: Please include a table or supplementary material listing all genomes used, including genome size, annotation information, database IDs, and a completeness measure, such as BUSCO scores.

To enhance transparency, provide a supplementary table detailing the RNA-seq data used, including species, strains, replicates, number of reads, and sequencing technology.

Improve the explanation of the two approaches for ncRNA prediction. For the first approach, the database link is missing, and the scope of the database used is unclear. Specify the database size. Additionally, the tools and parameters used for the search are not well-documented and are not available in the GitHub repository (ncRNA_leish).

For the second approach, clarify how each tool was applied and the specific settings used.

The methodology for validating ncRNA predictions is not sufficiently detailed. Explicitly describe the parameters rather than referencing other publications. Provide parameters for tools such as Bowtie and HTSeqCount Additionally, mention the CPM filter used in the results section.

Comparative analysis of ncRNAs: On line130, specify the coverage used. The Gblocks parameters also need to be detailed.

The characterization of RNAi/miRNA should be reorganized, possibly by including subsections within the methodology for different genomes.

When identifying AGO1-like proteins, the authors used BLAST, but could an HMM-based search provide complementary insights?.

Reviewer #3: There are some methodological issues pointed in the comments presented in the next section.

**********

**Results**

-Does the analysis presented match the analysis plan?

-Are the results clearly and completely presented?

-Are the figures (Tables, Images) of sufficient quality for clarity?

Reviewer #1: Unfortunately, the answer to those questions is NO

Reviewer #2: The abundance of detected ncRNA families could be influenced by genome completeness or size. Consider discussing this in more detail.

Figure 1A: Highlight the species used for determining the transcription values described in Figure 1B.

The distribution of clusters generated by CD-HIT and analyzed in the RNAome is not fully clear. For example, 7.95% represents 196,000 ncRNAs, while 92.05% represents 76,000 ncRNAs. Are these dispensable ncRNAs supported by transcriptomic data?

A notable cluster is absent in L. sp. D974 in Figure 2. Could this be due to genome annotation or completeness issues?

l.236: Correct the phrase “In terms.”

l.257–266: This section would benefit from reorganization. First, describe the total number of DEG genes identified in each species. Then focus on L. braziliensis, highlighting common DEGs between stages and associated GO terms. Finally, present results from the other two species in a separate paragraph.

Lines 281–305: Improve the hierarchy of the Gene Ontology analysis similarly to the suggestion above.

Line 311: Clarify “In contrast.”

Figure 5: Consider a different representation of co-expression networks. The current labels (IDs) are unreadable. A simplified network diagram with circular nodes in two colors (without labels) could visually illustrate the differences in the number of coding genes and miRNA-like transcripts between species and improve the aesthetics of the figure.

Figure 6: Similarly, parts A, B, and C in this figure are not informative in their current form. Consider alternative visualizations.

Line 407: Please revise the phrasing for clarity.

In the phylogeny presented in Fig.suppl. 1, some nodes can be rotated to better show the association between the phylogeny and the dendogram. Improved this figure is even interesting to show as the main figure.

Reviewer #3: In the first section “Dissecting the repertoire of non-coding RNAs across Leishmania spp.” the authors used computational combined approaches that includes covariance model comparisons of known RNA families, sequence similarity searches from public databases, and RNA-sequencing datasets to map the ncRNA repertoire across 26 genomes from 16 Leishmania species.

The analysis was based in two databases, NRDR/NR2 and Rfam v14, and the proportion of miRNA, siRNA and piRNA seems to be too high and too homogeneous across Leishmania species (Fig1A). There seems to be a bias that enriches the classes linked to the RNAi pathway, which only exists in the Viannia subgenus. It seems to this reviewer that the analysis may be biased by the databases used. I consider necessary that the authors use the annotation already available for well-known structural ncRNAs, such as rRNA and tRNA, in the genome of L. major (best annotated genome) to check for the proportion of these families to confirm their prediction.

Data presented in Fig1C is confusing. The authors used different scales in each panel making it difficult to compare, and the scales used do not help, for the few tick markers. Also, it calls the attention that in all families presented, the L. braziliensis levels are always lower even in the miRNA and piRNA families, which seems to be a contradiction, reinforcing the strangeness based on what is known about this machinery in Leishmania. It is also unexpected the low rRNA abundance, I guess that this would be true if the transcriptomes used as the source of the analysis were rRNA depleted. But the authors fail to indicate that and the presentation of levels of rRNAs using a rRNA depleted library, for their purposes, makes no biological sense and is misleading.

Despite the putative ‘RNA families’ bias inserted by the first steps of the computational approaches (as abovementioned) to identify represented RNA families, data analysis and corresponding representation in the section “The Leishmania spp. pan-RNAome: ncRNAs conservation across 26 genomes” is novel and brings information on sequence conservation of the putative ncRNAs with an interesting hierarchy of core/accessory/unique transcripts identified. In the lines 249-255 the considerations made on the phylogenetic/clusterization results are noteworthy as this analysis may be indicative of similar evolutionary patterns for coding and non-protein coding genes, which could have been further explored. Moreover, the authors mention the data 'en passant' in the discussion and it seems to this reviewer that these data deserve a more thorough discussion. It would be worthy for the authors to consider highlighting the data in the discussion.

In the section “Stage-specific differential expression of coding genes and ncRNAs” it is very difficult for the reader to follow the information presented in Figure 3 and accompanying text. Difficult to figure out the numbers presented, the text is not clear, and figure legend doesn’t help. The figure and text must be profoundly modified. In addition, authors inform that they have analysed DEG in L. donovani, L. major and L. braziliensis. L. donovani and L. major are presented in the supplementary file but in a distinct form, making any comparative analysis troublesome. The reader must go to each figure to understand and compare the numbers presented. The L. braziliensis data (Fig3A) presentation is very confusing.

Besides, the GO analysis presented lacks important information; the parent/children relationship of the terms is not discussed, inserting a bias to the figure as presented. In addition, p-values indicate that many of the GO enrichments are not soundly supported by the statistics. In the case of the metacyclics, none of the GOs pointed as enriched have a sound statistics support, and the number of genes per GO is very low. None of this is discussed or carefully presented. We suggest the authors to use a tool to make the parent/children relationship clear (AmiGO 2 > Tools & Resources > Visualization > GO IDs or advanced format (Paste json file) > Use advanced format > Output type: SVG > Visualize) and to conduct the reader on the significance of data as presented.

The section “Co-expression network analysis identifies developmental stages-associated gene modules in Leishmania spp.” needs major adjustments:

1. This reviewer couldn’t understand how the network was constructed and how the modules were associated with each stage, the methodology must be better explained (WGCNA and GS and MM Scores used to determine correlations must be included in the methodology). It is possible that I did not understand because I do not work with network construction, but this will be true for most of the readers. Furthermore, the construction of networks and correlations are central to the next analyses conducted, so it is essential that these aspects are clearly presented.

2. Supplementary Fig 4 was not included, thus, we have only the L. braziliensis heatmap. This reviewer considers that after explaining rational and approaches for these results, the heatmaps (L. braziliensis, L. donovani and L. major) should be all part of supplementary material.

When we get to the section “Detection of potential miRNA-like involved in amastigote life cycle in Leishmania parasites”, the authors start (line 337) by stating that To assess the possible functions of the identified miRNA-like in amastigote developmental stages, DE mRNAs and miRNAs-like RNAs were selected to filter the co-expression network corresponding to this life cycle stage in L.braziliensis and L. donovani.

The idea of filtering the co-expression network in a specific life-stage is interesting as with the filtering authors would avoid the difficulties of GO terms. Nevertheless, the filtering procedure is not clearly explained; the source of the numbers of DE mRNAs from L. braziliensis and L. donovani are not well-defined. What was the source used to reach 89 and 206 amastigotes preferentially expressed genes (mRNAs) in L. braziliensis and L. donovani? The numbers do not match with numbers from Fig 3 and if they come from articles published it must be indicated. Also, authors state (line 344) that 206 protein coding genes are exclusively expressed in amastigotes which is a very strong statement, given that few genes are known to be ‘exclusive’ of a given stage in these parasites, most of the DEGs in Leishmania are preferentially expressed but not “exclusive” of a given life stage.

Next, the authors apply a further filtering step (line 363) - Through the guilty by association functional annotation approach, we predicted the possible function of the top miRNA like that participates in the amastigote developmental stage in L. braziliensis and L. donovani. - with another methodology which is not even referred in the methods section.

Also, to the GO enrichment analyses presented in Figures 5 and 6 this reviewer extends the critique raised over similar data shown in Fig 3. As mentioned above: the parent/children relationship of the terms are not discussed, inserting a bias to the figure as presented. In addition, p-values indicate that many of the GO enrichments are not soundly supported by the statistics.

In the last results section “Identification of putative RNA interference pathway in Leishmania genomes” there is basically no novelties. That RNAi pathway is complete in Leishmania species of the subgenus Viannia is known since 2007 (reference 35, which is listed but not cited in the text). After discovery of the RNAi machinery in L. braziliensis, other authors explored functionally the pathway. Thus, it seems to this reviewer that the section and Figure 7 contains nothing that has not been explored and presented before. The authors should present in the results section only the new information highlighting what is new compared to what is publicly available.

In addition, Fig7D brings expression results that are misleading. For instance: the first panel of Fig7D presents AGO1 expression in L. major and L. donovani, but it is known that there are only remnants of AGO1 in Leishmania subgenus. So, what is the purpose of presenting the levels of expression of a gene known to be non-functional? What is the purpose of panel D? The form the authors present the data on expression of the other genes included in the same panel is also misleading; just to cite one, Exportin 1 is not an exclusive RNAi pathway protein. Exportin 1 has been studied by others and it has been shown, for instance, that it mediates nuclear export of SLRNA. So, what is the reason for the authors to present levels of expression of different genes with an unclear or unproved link to the RNAi pathway, which is, in addition, non-functional in the species of subgenus Leishmania?

Thus, in conclusion, this last topic should be either deleted or profoundly modified to show and highlight something new.

**********

**Conclusions**

-Are the conclusions supported by the data presented?

-Are the limitations of analysis clearly described?

-Do the authors discuss how these data can be helpful to advance our understanding of the topic under study?

-Is public health relevance addressed?

Reviewer #1: -Are the conclusions supported by the data presented? NO

Reviewer #2: Not provided

Reviewer #3: Some of the conclusions are not supported by the data presented (as noted in the comments above). If the critiques raised regarding certain analyses, results, and data presentation are accepted, the conclusions and interpretations drawn from these findings will need to be revised. The authors should ensure that their conclusions align more closely with the data presented and address the potential biases and limitations discussed. Clearer justification and contextualization of their results are essential to strengthen the manuscript’s overall validity and impact.

**********

**Editorial and Data Presentation Modifications?**

Use this section for editorial suggestions as well as relatively minor modifications of existing data that would enhance clarity. If the only modifications needed are minor and/or editorial, you may wish to recommend “Minor Revision” or “Accept”.

Reviewer #1: (No Response)

Reviewer #2: l.53: Replace “macrophage” with “cells (neutrophils, macrophages, and dendritic cells).”

l.88: The phrase “either conserved or unique among these species” seems redundant. Could the authors clarify the intended point?

Reviewer #3: Some typos were noticed but I did not highlight them at this point.

**********

**Summary and General Comments**

Use this section to provide overall comments, discuss strengths/weaknesses of the study, novelty, significance, general execution and scholarship. You may also include additional comments for the author, including concerns about dual publication, research ethics, or publication ethics. If requesting major revision, please articulate the new experiments that are needed.

Reviewer #1: The main objective of this study was to determine the non-coding RNAs (ncRNAs) repertoire in several Leishmania species. For this purpose, the authors used publicly available data consisting of genomic sequences and RNA-seq Illumina reads. The authors applied several bioinformatics tools and generated many results. However, the design of the study and methodological details need to be better explained; otherwise, the relevance of the results would be questionable.

Main points.

1. Clarification on how the authors predict the repertoire of ncRNAs (section 2.2.) is mandatory. Reference 58 describes a database created by compilation of non-coding RNA (ncRNAs) classes described in organisms of different kingdoms. Questions:

i) How ncRNAs were identified (Blast, structural features, etc.)?

ii) Do the authors know if the identified ncRNAs are real RNA molecules in Leishmania?

2. Figure 1 and accompanying text in the manuscript. Apart from rRNAs and tRNAs, what were the structural features used to define each category of ncRNAs? Most of the ncRNAs are within the groups “unclassified” and “others”. However, how the authors deduce they are ncRNAs is unclear. Without a clear explanation, their existence might be questionable.

3. In the legend to figure 1, it is read: ncRNAs gene prediction was validated by transcriptional evidence using RNA-seq analysis. What the evidence was (the existence of RNA-seq reads or the existence of annotated transcripts)? This is another relevant question to be clarified. The sole existence of reads is not a demonstration that the predicted ncRNAs exist such as annotated in this study.

4. Line 222. Please, specify the difference between dispensable ncRNAs and accessory-RNAome.

5. Line 242. It is read: These Viannia-specific clusters were primarily annotated as gene expression regulatory ncRNAs. What criteria served to define "gene expression regulatory ncRNAs"? Please, comment on it.

6. Lines 364-365. What is the meaning of "the LbrM2903_20.1_984445-984477_miRNA miRNA-like gene was co-expressed with 40 coding genes"? How co-expression was determined?

7. Although the manuscript includes a quite large list of references, there are relevant articles missed by the authors. Examples:

- Freitas Castro, F., Ruy, P. C., Nogueira Zeviani, K., Freitas Santos, R., Simoes Toledo, J. and Kaysel Cruz, A. (2017). Evidence of putative non-coding RNAs from Leishmania untranslated regions. Mol Biochem Parasitol 214, 69–74. doi: 10.1016/j.molbiopara.2017.04.002.

- Torres, F., Arias-Carrasco, R., Caris-Maldonado, J. C., Barral, A., Maracaja-Coutinho, V. and De Queiroz, A. T. L. (2017). LeishDB: a database of coding gene annotation and non-coding RNAs in Leishmania braziliensis. Database (Oxford) 2017, bax047. doi: 10.1093/database/bax047.

Detailed comparisons between the data reported in these studies (and others cited in the manuscript) and the results generated in this study merit being carried out.

Reviewer #2: This manuscript explores the role of non-coding RNAs (ncRNAs) in the development and pathogenesis of Leishmania spp., the protozoan parasites responsible for leishmaniasis. The study characterizes the ncRNA repertoire across 26 strains from 16 different Leishmania species, identifying both conserved and unique ncRNA clusters. It also analyzes expression patterns in relation to coding genes, revealing the significant involvement of ncRNAs in regulating parasite development and survival. Notably, several miRNA-like transcripts were found to be co-expressed with coding genes associated with starvation, survival, and histone modification. Additionally, the authors investigate the presence of an RNA interference (RNAi) pathway in Leishmania, identifying some components of the pathway while suggesting the possibility of a non-canonical RNAi mechanism due to the absence of key canonical components.

Despite its significant contribution to the understanding of ncRNAs—particularly miRNA-like RNAs—and the potential identification of non-canonical RNAi pathways, the article could benefit from improvements in the methodological rigor and a more structured and hierarchical presentation of the results. Specifically, the quality and completeness of the datasets used for inference are not sufficiently discussed.

Overall, the manuscript is well-presented; however, given its focus on a comprehensive in silico analysis, it would benefit from stronger support in terms of data quality and applied methodologies. The presentation of a large amount of data and comparisons makes the narrative difficult to follow at times. A clearer hierarchical structure within sections and the use of shorter sentences would improve the readability and coherence of the results.

Reviewer #3: The authors aimed to identify ncRNAs from Leishmania spp. to gain insights into the RNAome (repertoire of ncRNAs) of this protozoan genus by analyzing a large number of strains and species. They performed computational analyses to investigate co-expression relationships between coding genes and ncRNAs expressed in all stages of Leishmania braziliensis, L. donovani and L. major. While the study is commendable for incorporating a substantial number of Leishmania strains and species (26 strains representing 16 different species) and different levels of supporting data, there are significant limitations in the work that currently prevent its recommendation for publication.

**********

PLOS authors have the option to publish the peer review history of their article (what does this mean? ). If published, this will include your full peer review and any attached files.

If you choose “no”, your identity will remain anonymous but your review may still be made public.

**Do you want your identity to be public for this peer review?** For information about this choice, including consent withdrawal, please see our Privacy Policy .

Reviewer #1: No

Reviewer #2: No

Reviewer #3: No

[NOTE: If reviewer comments were submitted as an attachment file, they will be attached to this email and accessible via the submission site. Please log into your account, locate the manuscript record, and check for the action link "View Attachments". If this link does not appear, there are no attachment files.] **Figure resubmission:**While revising your submission, please upload your figure files to the Preflight Analysis and Conversion Engine (PACE) digital diagnostic tool, https://pacev2.apexcovantage.com/. PACE helps ensure that figures meet PLOS requirements. To use PACE, you must first register as a user. Registration is free. Then, login and navigate to the UPLOAD tab, where you will find detailed instructions on how to use the tool. If you encounter any issues or have any questions when using PACE, please email PLOS at figures@plos.org. Please note that Supporting Information files do not need this step. If there are other versions of figure files still present in your submission file inventory at resubmission, please replace them with the PACE-processed versions. **Reproducibility:**To enhance the reproducibility of your results, we recommend that authors of applicable studies deposit laboratory protocols in protocols.io, where a protocol can be assigned its own identifier (DOI) such that it can be cited independently in the future. Additionally, PLOS ONE offers an option to publish peer-reviewed clinical study protocols. Read more information on sharing protocols at https://plos.org/protocols?utm_medium=editorial-email&utm_source=authorletters&utm_campaign=protocols

# Author comment: Author response to Decision Letter 0

**DOI:** 10.1371/journal.pntd.0013108.r003

14 Mar 2025

Attachment Submitted filename: rebuttal_letter_PlosNTD.pdf

# Peer review: Decision Letter 1

**DOI:** 10.1371/journal.pntd.0013108.r004

**Authors:** Claudia Brodskyn

5 May 2025

Dear Dr. Martin,

We are pleased to inform you that your manuscript 'Comparative and systems analyses of Leishmania spp. non-coding RNAs through developmental stages' has been provisionally accepted for publication in PLOS Neglected Tropical Diseases.

Before your manuscript can be formally accepted you will need to complete some formatting changes, which you will receive in a follow up email. A member of our team will be in touch with a set of requests.

Please note that your manuscript will not be scheduled for publication until you have made the required changes, so a swift response is appreciated.

IMPORTANT: The editorial review process is now complete. PLOS will only permit corrections to spelling, formatting or significant scientific errors from this point onwards. Requests for major changes, or any which affect the scientific understanding of your work, will cause delays to the publication date of your manuscript.

Should you, your institution's press office or the journal office choose to press release your paper, you will automatically be opted out of early publication. We ask that you notify us now if you or your institution is planning to press release the article. All press must be co-ordinated with PLOS.

Thank you again for supporting Open Access publishing; we are looking forward to publishing your work in PLOS Neglected Tropical Diseases.

Best regards,

Claudia Ida Brodskyn

Section Editor

PLOS Neglected Tropical Diseases

Claudia Brodskyn

Section Editor

PLOS Neglected Tropical Diseases

Shaden Kamhawi

co-Editor-in-Chief

PLOS Neglected Tropical Diseases

orcid.org/0000-0003-4304-636XX

Paul Brindley

co-Editor-in-Chief

PLOS Neglected Tropical Diseases

orcid.org/0000-0003-1765-0002

***********************************************************

Reviewer's Responses to Questions

**Key Review Criteria Required for Acceptance?**

As you describe the new analyses required for acceptance, please consider the following:

**Methods**

-Are the objectives of the study clearly articulated with a clear testable hypothesis stated?

-Is the study design appropriate to address the stated objectives?

-Is the population clearly described and appropriate for the hypothesis being tested?

-Is the sample size sufficient to ensure adequate power to address the hypothesis being tested?

-Were correct statistical analysis used to support conclusions?

-Are there concerns about ethical or regulatory requirements being met?

Reviewer #1: -

Reviewer #3: The authors have significantly revised the original manuscript, addressed the reviewers' comments, and the methodology is now complete and clearly presented. Therefore, I positively acknowledge all applicable items in the methods section.

**********

**Results**

-Does the analysis presented match the analysis plan?

-Are the results clearly and completely presented?

-Are the figures (Tables, Images) of sufficient quality for clarity?

Reviewer #1: -

Reviewer #3: The authors have significantly revised the original manuscript, addressed the reviewers' comments. The results section has been significantly improved, and the same positive appreciation applies. Figures and tables are improved, clearer and more informative.

**********

**Conclusions**

-Are the conclusions supported by the data presented?

-Are the limitations of analysis clearly described?

-Do the authors discuss how these data can be helpful to advance our understanding of the topic under study?

-Is public health relevance addressed?

Reviewer #1: -

Reviewer #3: The authors have also significantly revised the Discussion section, establishing appropriate connections between the data/results and the possible conclusions. Additionally, they have further explored some of the data, enhancing the overall significance of the contribution.

**********

**Editorial and Data Presentation Modifications?**

Use this section for editorial suggestions as well as relatively minor modifications of existing data that would enhance clarity. If the only modifications needed are minor and/or editorial, you may wish to recommend “Minor Revision” or “Accept”.

Reviewer #1: -

Reviewer #3: I have only three minor-minor comments, that could be considered editorial modifications.

Lines 97-99 -

The authors should tone down the sentence, as it sounds like an overstatement.

Furthermore, this work enhances our comprehension of the molecular biology of Leishmania and opens new ways for the development of strategies, aimed at targeting ncRNA-driven mechanisms in the fight against leishmaniasis.

Lines 135-138 -

I encountered difficulties with the flow of the text, for which the AI proposed an alternative construction:

This step enables us to perform local-to-local alignments, thereby predicting ncRNAs with ≥80% sequence similarity. Consequently, it also allows us to identify the specific genomic regions where sequence homology between ncRNAs and Leishmania genomes is detected.

Line 369 – a correction to be made: verbal concordance

These clusters was mostly made up of unclassified ncRNAs

**********

**Summary and General Comments**

Use this section to provide overall comments, discuss strengths/weaknesses of the study, novelty, significance, general execution and scholarship. You may also include additional comments for the author, including concerns about dual publication, research ethics, or publication ethics. If requesting major revision, please articulate the new experiments that are needed.

Reviewer #1: After reading the revised manuscript and the authors’ responses to my previous comments, I feel that the authors have sufficiently addressed the suggestions. Thus, I recommend that the manuscript be accepted at this stage.

Reviewer #3: I commend the authors for their thorough work and substantial revision of the manuscript. The reviewers' comments have been addressed point by point, resulting in a significantly improved version of the original submission. The revised manuscript represents a coherent and valuable contribution to the field.

**********

PLOS authors have the option to publish the peer review history of their article (what does this mean? ). If published, this will include your full peer review and any attached files.

If you choose “no”, your identity will remain anonymous but your review may still be made public.

**Do you want your identity to be public for this peer review?** For information about this choice, including consent withdrawal, please see our Privacy Policy .

Reviewer #1: No

Reviewer #3: **Yes: ** Angela K. Cruz

# Editor report: Acceptance letter

**DOI:** 10.1371/journal.pntd.0013108.r005

**Authors:** Claudia Brodskyn

Dear Dr. Martin,

We are delighted to inform you that your manuscript, "Comparative and systems analyses of Leishmania spp. non-coding RNAs through developmental stages," has been formally accepted for publication in PLOS Neglected Tropical Diseases.

We have now passed your article onto the PLOS Production Department who will complete the rest of the publication process. All authors will receive a confirmation email upon publication.

The corresponding author will soon be receiving a typeset proof for review, to ensure errors have not been introduced during production. Please review the PDF proof of your manuscript carefully, as this is the last chance to correct any scientific or type-setting errors. Please note that major changes, or those which affect the scientific understanding of the work, will likely cause delays to the publication date of your manuscript. Note: Proofs for Front Matter articles (Editorial, Viewpoint, Symposium, Review, etc...) are generated on a different schedule and may not be made available as quickly.

Soon after your final files are uploaded, the early version of your manuscript will be published online unless you opted out of this process. The date of the early version will be your article's publication date. The final article will be published to the same URL, and all versions of the paper will be accessible to readers.

Thank you again for supporting open-access publishing; we are looking forward to publishing your work in PLOS Neglected Tropical Diseases.

Best regards,

Shaden Kamhawi

co-Editor-in-Chief

PLOS Neglected Tropical Diseases

Paul Brindley

co-Editor-in-Chief

PLOS Neglected Tropical Diseases
